# Deep Eutectic Solvent Extraction of Heteratisine from *Aconitum tanguticum*: COSMO-RS Screening, RSM Optimization and Machine Learning Comparison

**DOI:** 10.3390/plants15213362

**Published:** 2026-10-29

**Authors:** Ruixi Gao, Haishuo Huang, Xinyang Li, Junjie Ma, Jiawei Dai, Yuchen Lei, Liqun Ma, Awais Ihsan, Qien Li, Jun Li, Qupei Danzeng, Xianju Huang

**Affiliations:** 1 School of Pharmaceutical Sciences, South-Central Minzu University, Wuhan 430074, China; 2023021@mail.scuec.edu.cn (R.G.);; 2 College of Life Sciences, South-Central Minzu University, Wuhan 430074, China; 3 Department of Biosciences, COMSATS University Islamabad, Sahiwal Campus, Sahiwal 57000, Pakistan; 4 Tibetan Medical College, Qinghai University, Xining 810016, China; 5 Department of Tibetan Pharmacy, Xizang University of Tibetan Medicine, Lhasa 850000, China; 6 Hubei International Science and Technology Cooperation Base (SH2311), South-Central Minzu University, Wuhan 430074, China

**Keywords:** *Aconitum tanguticum* (Maxim.) Stapf, heteratisine, deep eutectic solvent, COSMO-RS, machine learning

## Abstract

*Aconitum tanguticum* (Maxim.) Stapf is a Tibetan medicinal plant with low toxicity. To obtain the major alkaloid heteratisine (HTS) for bioactivity evaluation, this study established a method combining deep eutectic solvent (DES) with ultrasound-assisted extraction. COSMO-RS screening identified menthol-octanoic acid (1:2) as the optimal solvent, giving a content of 0.321 mg/g, approximately 9.17 times that of ethanol. Extraction optimization was performed via response surface methodology on a 29-run Box–Behnken design, providing a validated optimum of 0.499 mg/g at 256.8 W, 30.5 min, 1:30.3 g/mL, and 39.7 °C. The fitting behavior of three machine learning models and quadratic polynomial regression (QPR) was compared on a 75-run non-structured dataset. XGBoost and GPR achieved high in-sample fitting performance (R^2^ = 0.983 and 0.994), whereas QPR and Random Forest showed limited fitting (0.529 and 0.546). Condition-grouped nested validation demonstrated that none of the models could extrapolate beyond the observed dataset. Thus, the ML analyses were framed as in-sample diagnostic tools, with GPR offering uncertainty estimates to identify data-sparse regions. In HEK293T-TRPV1 cells, HTS pretreatment reduced capsaicin-induced Ca^2+^ signaling. In general, this study provides a DES-based extraction approach and a multi-model comparison framework for the utilization of *A. tanguticum*.

## 1. Introduction

The genus *Aconitum* is traditionally used for pain relief, and its diterpenoid alkaloids have been reported to inhibit the transient receptor potential vanilloid subtype 1 (TRPV1) channel, a peripheral node involved in pain signal transduction [1,2]. Among its species, *Aconitum tanguticum* (Maxim.) Stapf (Tibetan name “Bangga”) is distinguished by its relatively low toxicity compared with conventional *Aconitum* species and abundance of lactone-type diterpenoid alkaloids [3]. As one of its major alkaloids, heteratisine (HTS; mouse intraperitoneal injection LD_50_ of 430 mg/kg, approximately 1400-fold less toxic than aconitine) accounts for 0.05% of the dried plant weight, a level comparable to the determination content required for *Aconitum* species in the Chinese Pharmacopoeia, with reported antiarrhythmic and anticholinesterase activities [4,5,6]. Therefore, investigation of the HTS extraction can provide a basis for its large-scale isolation, content determination and bioactivity testing. Current methods for alkaloid extraction often use chloroform or ethanol, with ultrasound-assisted extraction (UAE) being an efficient procedure [7,8]. However, ethanol poorly dissolves the weakly polar HTS, and chloroform is effective but genotoxic, making it unsuitable for safe extraction [9]. Thus, an efficient extraction method for HTS is needed.

Deep eutectic solvents (DES) are mixtures formed by hydrogen bond acceptors (HBAs) and donors (HBDs). They are non-volatile, easy to prepare and tunable, and have been successfully used for the extraction of alkaloids, such as vinblastine and sinomenine [8,10]. Despite these advantages, the numerous possible DES combinations and variable extraction factors (power, time, solid–liquid ratio, and temperature) render exhaustive experimental screening impractical, thus calling for both solvent screening and extraction optimization. To address this, the conductor-like screening model for real solvents (COSMO-RS) can be employed to predict solute-solvent compatibility and prescreen suitable DES candidates [11]. COSMO-RS calculates molecular surface charge density distributions via density functional theory (σ-profiles) and converts them into interaction energies (σ-potentials) through statistical thermodynamics, allowing the prediction of infinite dilution activity coefficients (lnγ^∞^) and solubility. Using this approach, DES candidates can be selected without experiments, which improves the efficiency of extraction processes [12,13].

In extraction modeling, quadratic polynomial regression (QPR) is widely used to model response surfaces, with response surface methodology (RSM) being its classical implementation under structured experimental designs such as Box–Behnken and central composite designs (BBD and CCD) [14]. However, the validity of QPR depends on proper model specification, full rank of the design matrix, and adequate sampling to estimate curvature. When these conditions are not met, for example, when data are collected incrementally across stages or when full factorial control is not feasible, the reliability of QPR-based estimation will decline sharply [15]. In such cases, machine learning (ML) can provide alternative analytical approaches, as it extracts patterns from data without relying on a fixed equation form. Among ML approaches, eXtreme Gradient Boosting (XGBoost) combined with a genetic algorithm (GA) can explore high-content candidate conditions, Random Forest (RF) can identify important process factors via feature importance ranking, and Gaussian Process Regression (GPR) can provide probabilistic estimates with quantified uncertainty [16,17,18]. Since these methods have different strengths, comparing their fitting performance on the same dataset can help reveal their respective contributions within the data-covered range.

In this study, we evaluated different DES systems for HTS extraction using ethanol and chloroform as controls. These DES systems included a zwitterionic type (betaine-propylene glycol, Bet-PG), quaternary ammonium salts (choline chloride with lactic acid, glycerol, propylene glycol and urea, ChCl-LA, Gly, PG and Urea), an organic acid type (lactic acid-glycerol, LA-Gly) and hydrophobic DES systems (lactic acid-menthol, thymol-menthol, and menthol-octanoic acid, LA-Men, Thy-Men, Men-Oct). COSMO-RS was used to predict the solubility and lnγ∞ of HTS in these DES systems, and the predicted candidates were experimentally validated. After identifying the best DES, single-factor experiments were performed to determine the appropriate ranges of ultrasonic power, time, solid–liquid ratio and temperature. Process optimization was then carried out via RSM on a 29-run BBD, and the optimal conditions were scaled up for preparative extraction, HPLC purification and NMR identification of HTS (Figure S1). In parallel, a 75-run dataset, covering a broader range of factor combinations in corner-point regions, was used to compare the estimation performance of QPR, XGBoost, RF, and GPR. To preliminarily evaluate the bioactivity of HTS, two established cell-based assays were employed: lipopolysaccharide (LPS)-stimulated RAW264.7 macrophages for assessing inhibition of inflammatory mediator release, and capsaicin (CAP)-induced Ca^2+^ signaling in HEK293T-TRPV1 cells [19,20].

## 2. Results

### 2.1. COSMO-RS Guided DES Screening

#### 2.1.1. Solubility Analysis Based on σ-Profile and σ-Potential

The σ-profile and σ-potential are two representative parameters of COSMO-RS theory, which quantify the statistical distribution of the polarization charge density on a molecular surface and characterize the interaction affinity between the solute and solvent at each charge density region, respectively [21]. According to the standard COSMO-RS convention, the σ-axis is divided into three regions: HBD (σ < −0.0082 e/Å^−2^), nonpolar (−0.0082 e/Å^−2^ ≤ σ ≤ +0.0082 e/Å^−2^), and HBA (σ > +0.0082 e/Å^−2^) [22]. The σ-profile of HTS (Figure S2) consists of a near-zero charge region (σ ≈ 0) occupying approximately 75% of the molecular surface, together with negative (σ < 0) and positive (σ > 0) regions accounting for the remaining 25%. This indicates that the surface of HTS is predominantly nonpolar, rendering it overall hydrophobic. Its σ-potential curve (Figure S3) reveals that in the negative region (σ < 0), μ(σ) decreases with increasing charge density, reaching approximately −0.8 kcal/molÅ^−2^ (σ = −0.03 e/Å^−2^), indicating an affinity for HBD solvents such as hydroxyl-containing solvents. In the positive region (σ > 0), μ(σ) is negative for σ below 0.01 e/Å^−2^, then crosses zero (σ ≈ 0.01 e/Å^−2^) and increases to a positive maximum of approximately +0.01 kcal/molÅ^−2^ (σ ≈ 0.02 e/Å^−2^), indicating repulsion toward high charge density HBA sites (quaternary ammonium salts or choline-based moieties) [23]. In particular, the carboxyl group can act as both an HBA (for the carbonyl oxygen) and an HBD (for the hydroxyl hydrogen), indicating dual roles in different DES systems [24]. Therefore, DES systems containing carboxyl groups can further promote dissolution (ChCl-LA, LA-Gly, LA-Men, and Men-Oct) and increasing the molar ratio of carboxyl-bearing components correspondingly improves the calculated solubility of HTS (Table 1).

**Figure 1:**
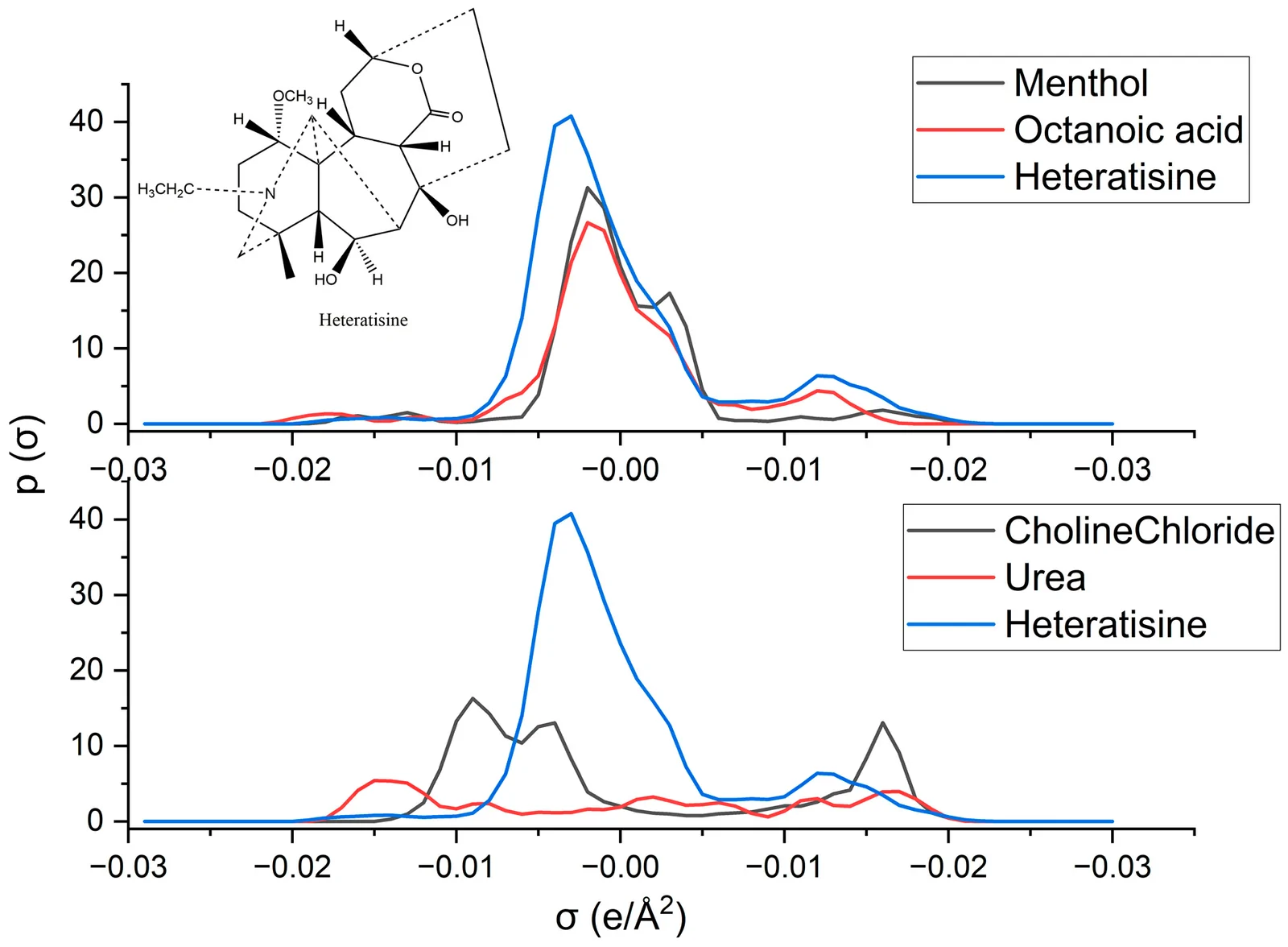
Chemical structure of heteratisine and its σ-profiles in menthol-octanoic acid and choline chloride-urea.

A COSMO-RS analysis was also performed on the representative strongly polar DES, ChCl-Urea. The σ-potential curves for ChCl and Urea (Figure S4) both showed negative values in the HBD region, indicating a high affinity for HBD groups. Moreover, their σ-potentials also exhibited highly negative values in the HBA region, demonstrating a strong HBA ability. This combination results in a high overall polarity of the DES. Consequently, a polarity mismatch arises with the predominantly hydrophobic σ-profile of HTS (Figure 1). This unfavorable interaction led to poor solvation stability and low calculated solubility (1.24 × 10^−6^, Table 1), which was consistent with its undetectable experimental content. Adjusting the HBD proportion (e.g., increasing LA or PG in ChCl-based systems) can moderately dilute the ChCl fraction to reduce the effective polarity; however, such dilution is insufficient to overcome the fundamental polar mismatch with the hydrophobic HTS surface.

#### 2.1.2. Evaluation of Extraction Solvent Performance

To quantify the solute-solvent affinity predicted by the σ-profile and σ-potential analyses, the COSMO-RS-derived infinite-dilution activity coefficient (lnγ^∞^) and calculated mass fraction solubility were employed (Table 1). The infinite dilution activity coefficient lnγ^∞^ reflects the strength of non-ideal solute-solvent interactions (more negative values indicate stronger interactions and more stable solvation), while the calculated mass fraction solubility is inversely related to lnγ^∞^ and serves as a direct indicator of solvation capacity [24]. Based on lnγ^∞^ and calculated solubility, the predicted solvents can be divided into three categories: high-efficiency, medium, and poor. The high-efficiency solvents (Men-Oct and CHCl_3_) both show relatively low lnγ^∞^ values (−2.342 and −4.194) and calculated solubility (0.01 and 0.06), indicating strong solute-solvent affinity. Among the tested solvents, Men-Oct provided the highest experimental extraction content (0.321 mg/g), which is consistent with its σ profile: the charge density distribution interval matches that of HTS (Figure 1), along with a lower probability density p(σ), indicating a less pronounced surface charge distribution but retaining the same polarity regime.

Among the medium solvents, LA-Men (0.106 mg/g), Thy-Men (0.062 mg/g), and EtOH exhibited lnγ^∞^ values of −1.739, −1.32, and −0.304, respectively, with calculated solubility values of 6.2 × 10^−3^, 3.579 × 10^−3^, and 4.36 × 10^−3^, respectively. The ineffective solvents (all ChCl-based and Be-based DES systems) possessed lnγ^∞^ values greater than 3.0, calculated solubilities lower than 10^−4^. In these systems, HTS was not detected by HPLC under the tested conditions, with experimental contents below the LOD (Figure S5). The trends of lnγ^∞^ and calculated solubility correlated with the polarity and functional groups of the HBD/HBA in each DES. When the HBA or HBD component is fixed (Bet-PG/ChCl-PG, or LA-Men/Thy-Men/Men-Oct), the weaker the polarity of the counterpart, the better the compatibility with HTS. This is exemplified by the hydrophobic series: replacing glycerol in LA-Gly (1:2) with menthol or thymol shifted lnγ^∞^ from 1.554 to −1.739 and −1.32, respectively, while substituting octanoic acid for lactic acid in the menthol-based system further lowered lnγ^∞^ to −2.342 and raised the calculated solubility to 0.01. In addition, DES systems containing carboxyl groups tend to show relatively lower lnγ^∞^ and higher solubility values, which is consistent with the analysis mentioned above. Thus, the Men-Oct DES was selected as the extraction solvent and used for the next optimization process.

### 2.2. Extraction Optimization and Model Comparison

#### 2.2.1. RSM Optimization

Prior to process optimization, the suitability of the HPLC method for the HTS content range was confirmed. The calibration curves were linear over injected amounts of 0.5–6 μg and 0.05–0.6 μg, with the regression equations Y = 7 × 10^6^ X + 52.094 (R^2^ = 0.9994) and Y = 7.02 × 10^6^ X + 51.518 (R^2^ = 0.9996), respectively. Acceptable precision was confirmed by standard solution repeatability (relative standard deviation, RSD, 0.87%), sample preparation repeatability (RSD 0.56%) and solution stability over 24 h (RSD 2.1%). Spike-and-recovery assays gave recoveries between 99.35% and 101.1% (RSD 1.68%). Following the described sample preparation procedures, the method was validated for HTS quantification in EtOH and Thy-Men extracts in the range of 0.01–0.05 mg/g, and for process investigation samples in the range of 0.1–0.5 mg/g. According to the signal-to-noise criteria of 3:1 for LOD and 10:1 for LOQ, the LOD and LOQ of HTS were determined to be 1.0 μg/mL and 3.3 μg/mL, respectively.

**Figure 2:**
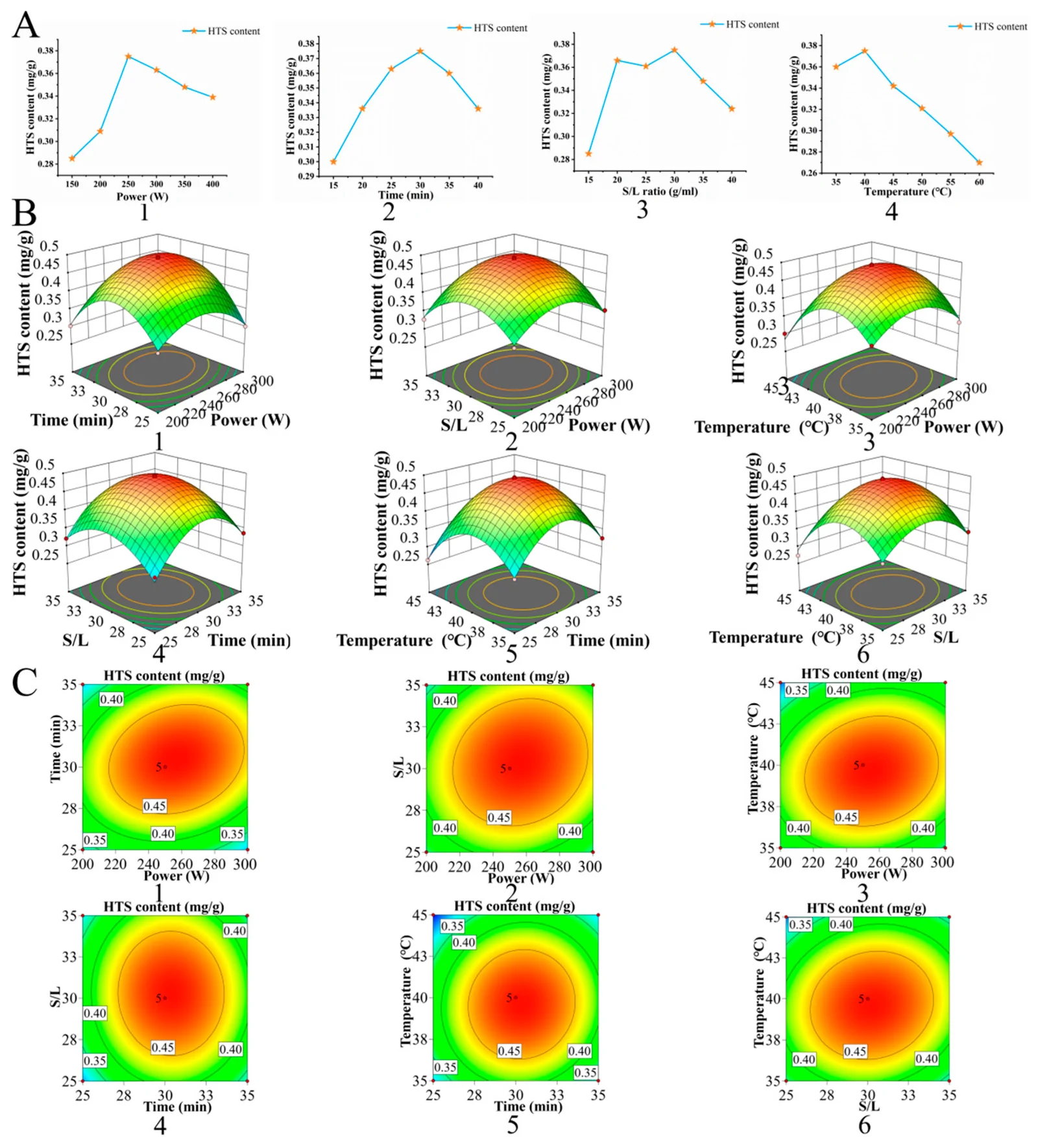
Single-factor (**A**) and RSM optimization (**B**,**C**) of ultrasound-assisted HTS extraction. Numbers 1–4 in (**A**) represent the single-factor experiments of power, time, S/L ratio, and temperature. Numbers 1–6 in (**B**,**C**) represent the two-factor interaction effects.

Single-factor results (Figure 2A) showed that ultrasonic power, time, solid–liquid ratio and extraction temperature each presented a trend of initial increase then decrease for HTS content. Among these, ultrasonic power contributed the most, followed by time, solid–liquid ratio and temperature. Ultrasonic power from 150 W to 250 W raised the content from 0.285 mg/g to 0.375 mg/g, but a further increase reduced the content. This was because moderate power enhanced cavitation and mechanical shearing to break cell walls and release HTS, whereas excessive power generated bubbles that blocked sound energy and caused local overheating, leading to compound degradation [25,26]. For extraction time, the content rose slowly from 0.3 mg/g at 15 min to 0.375 mg/g at 30 min, then dropped after 30 min due to saturation of mass transfer and possible decomposition of HTS and loss of solvent from sonication. The solid–liquid ratio displayed a double-peak pattern, with an initial content of 0.285 mg/g at 1:15 g/mL, a first peak of 0.366 mg/g at 1:20 g/mL, a second peak of 0.375 mg/g at 1:30 g/mL, and a decrease afterward, suggesting that an appropriate ratio improved the concentration gradient for mass transfer and used the dissolving power of DES for HTS, while too much solvent diluted the solute concentration and dispersed ultrasonic energy, thus lowering extraction efficiency. As a diterpene alkaloid with a complex structure and low thermal stability, HTS was limited by extraction temperature as a key factor. Increasing temperature from 35 °C to 40 °C elevated the content from 0.36 mg/g to 0.375 mg/g, but a further temperature rise led to a sharp drop. Excessive heat weakened ultrasonic cavitation and degraded the HTS structure, and this was consistent with the low extraction temperature conditions for alkaloids [27,28]. Single-factor experiments identified optimal levels of 250 W, 30 min, 1:30 g/mL, and 40 °C, which served as center points (0). Low (−1) and high (+1) levels were set at 200/300 W, 25/35 min, 1:25/1:35 g/mL, and 35/45 °C, respectively, corresponding to step sizes of ±50 W, ±5 min, ±5 ratio units, and ±5 °C. Intervals were chosen from curve inflection points and effective response ranges to ensure coverage of the region where response changed while avoiding incomplete extraction or severe degradation. The resulting ranges were then used for response surface optimization. Taking these factors as independent variables and the extraction content as the response (Table S4), a multiple quadratic regression equation was established as follows: Y = 0.4887 + 0.0145A + 0.0143B + 0.0084C − 0.0152D + 0.0266AB + 0.0174AC + 0.0206AD − 0.0020BC + 0.0086BD + 0.0156CD − 0.0619A^2^ − 0.0950B^2^ − 0.0699C^2^ − 0.0918D^2^, where Y represents the content and A, B, C, D are extraction power, time, solid–liquid ratio and temperature, respectively.

The ANOVA results (Table 2) indicated that the model was statistically significant, demonstrating the fit of the regression equation to the extraction data. The lack of fit was not significant, which confirmed that the model adequately fitted the data without missing important factors. Among the interaction terms (Figure 2B), AB (power and time), AC (power and solid–liquid ratio), AD (power and temperature), and CD (solid–liquid ratio and temperature) were significant (*p* < 0.05). Their contour plots (Figure 2C) showed non-parallel contours, indicating that the effect of one factor on extraction content depended on the level of the other factor [29]. In contrast, BC (time and solid–liquid ratio) and BD (time and temperature) were not significant (*p* > 0.05), suggesting that no significant interaction was detected within the tested range and that these factor pairs could be optimized with less concern for cross-interference. All linear terms affected HTS content, while all quadratic terms (A^2^, B^2^, C^2^ and D^2^) were significant (*p* < 0.0001). Significant linear and quadratic terms of C indicate a nonlinear effect of the solid–liquid ratio within the tested range, meaning that the optimal solid–liquid ratio lies within the tested range (1:25–1:35) and content decreases on either side. Based on the 29-run BBD, the optimal extraction parameters were: ultrasound power 256.8 W, time 30.5 min, solid-liquid ratio 1:30.3 g/mL and heating temperature 39.7 °C, with an estimated HTS content of 0.491 mg/g and desirability of 0.985. Relative to the HTS content obtained by chloroform extraction (0.55 mg/g), the optimized DES-based method gave 0.499 mg/g, corresponding to an extraction efficiency of approximately 90.7%. For the 200 g sample preparation, extraction under the optimized conditions followed by medium-pressure column chromatography and preparative HPLC afforded 62.3 mg HTS after vacuum drying. The overall recovery was calculated to be approximately 62.4% based on the HTS extracted under the optimized DES conditions. HPLC analysis showed a purity of >95%. After vacuum drying, no obvious residual solvent signals were observed above the NMR detection limit (0.1% *w*/*w*, equivalent to 1 mg/g), and the structure was confirmed by HR-ESI-MS and NMR (Figure S1).

#### 2.2.2. ML Model Performance Evaluation

**Figure 3:**
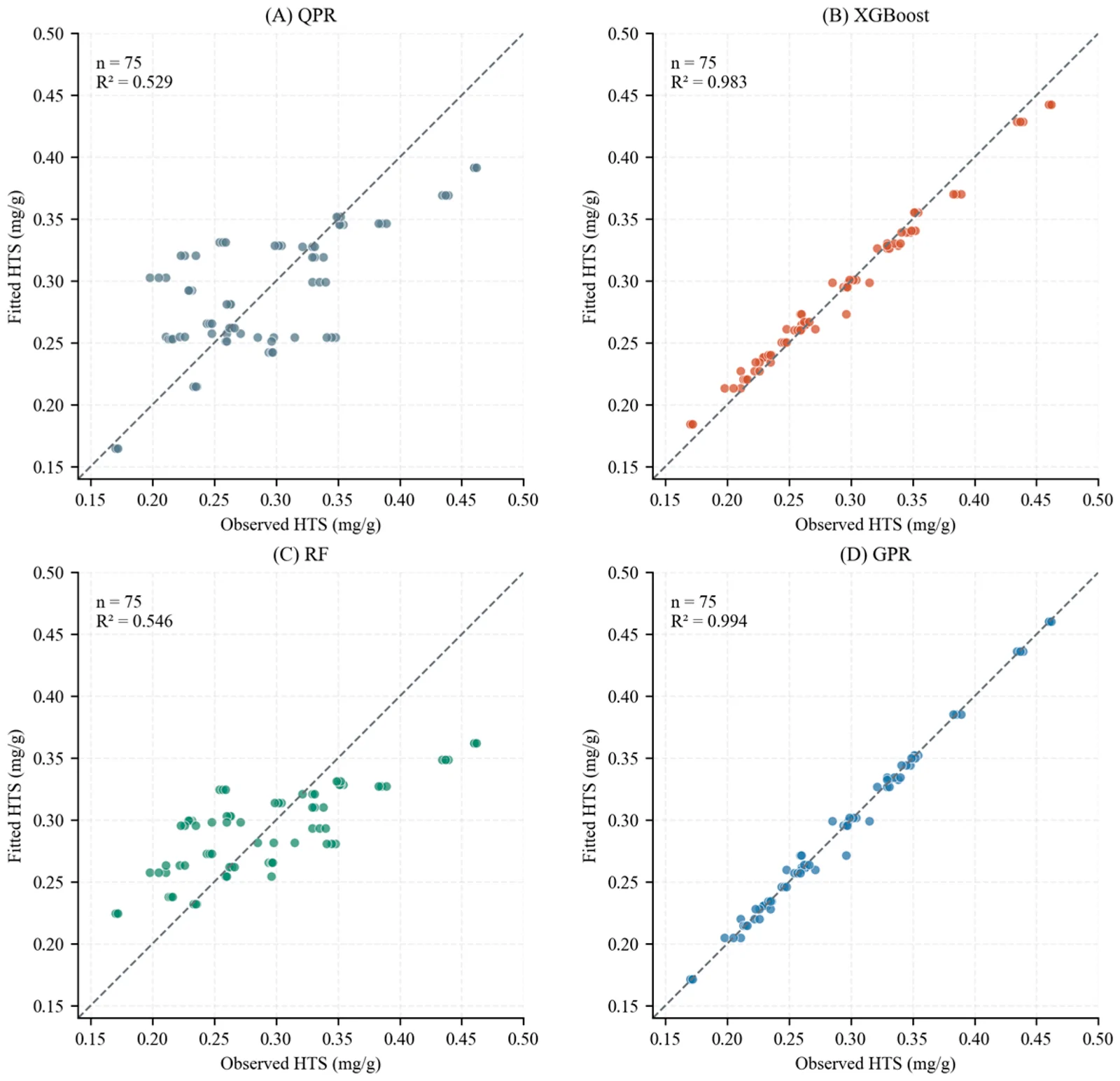
Scatter plots of estimated vs. measured HTS content for quadratic polynomial regression (QPR), extreme gradient boosting (XGBoost), random forest (RF), and Gaussian process regression (GPR).

In this study, modeling was used to evaluate the fitting behavior of different approaches on datasets with varying structural characteristics. Two datasets were employed: a 29-run BBD dataset (structured design space) to evaluate the fitting performance of four models, and a 75-run dataset (broader, less structured combinations) to assess estimation performance. Under unified selection rules involving identical hyperparameter search spaces and condition-grouped cross-validation (Tables S1–S6), all models on the 29-run structured BBD dataset achieved high and comparable in-sample fitting performance: RSM (QPR) 0.989, XGBoost 0.998, RF 0.969, and GPR 0.998 (Table S7). These results indicate that within the structured design, both RSM and ML methods achieve good fitting performance, making model choice less critical. However, on the 75-run dataset (Tables S8–S9), XGBoost and GPR still demonstrated high in-sample fitting performance (R^2^ = 0.983 and 0.994, respectively), whereas QPR and RF showed poor fitting, with R^2^ = 0.529 for QPR and 0.546 for RF (Figure 3). Detailed error metrics are provided in Table S10.

QPR performs poorly because in the encoded equation (Y = 0.3486 − 0.0005A + 0.0136B + 0.0087C − 0.0133D − 0.0497A^2^ − 0.0065B^2^ − 0.0262C^2^ + 0.0186AB + 0.0059AC + 0.0138AD − 0.0068BC − 0.0043BD − 0.0424CD), factor D (temperature) has only two levels (35 °C and 40 °C), D^2^ is collinear with −D and is omitted. Furthermore, the 75-run dataset is composed of corner-point combinations and lacks center and axial points. Such data geometry does not provide adequate sampling for independent estimation of a quadratic temperature effect, and the design matrix does not support the same model specification as the 29-run BBD. Regarding RF, cross-validation allowed a deeper tree (depth = 7) on the structured 29-run data to capture the well-defined response surface, achieving an R^2^ of 0.969. Conversely, to prevent overfitting on the non-structured 75-run data, the unified rule forced a restricted shallow tree (depth = 3, minimum 4 samples per leaf), limiting its in-sample capacity to an R^2^ of 0.546. The necessity of the adaptive strategy is further confirmed by a sensitivity check. When the fixed 75-run configuration was applied to the 29-run data, the R^2^ of the RF model dropped sharply to 0.331 (Table S11). Consequently, a single rigid parameter set cannot accommodate datasets with fundamentally different structural characteristics.

However, reliable extrapolation to conditions outside the experimental dataset requires validation. A condition-grouped nested validation was therefore performed using 60 training runs and 15 held-out runs while keeping all replicates of a condition together (Table S12). The mean outer-held-out R^2^ values were negative for all models (QPR −1.432, XGBoost −0.280, RF −0.068, and GPR −0.233; Table S13). Negative values demonstrate that none of the ML models can reliably predict conditions not covered by the observed data. Consequently, all ML-derived results are strictly restricted to in-sample fitting descriptions without predictive or process-design claims.

**Figure 4:**
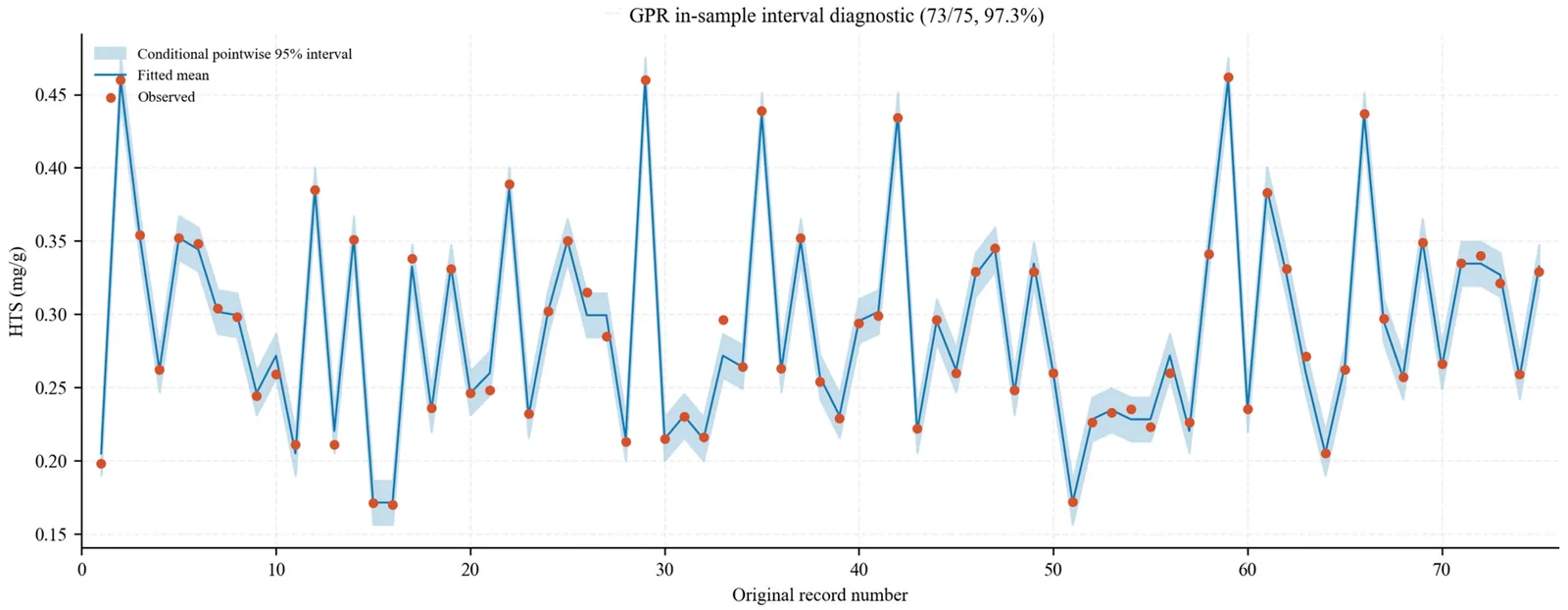
Gaussian Process Regression (GPR) fitted means and 95% observation intervals for 75 runs.

Among these models, GPR provides a unique diagnostic value through uncertainty quantification. It yields conditional pointwise 95% observation intervals, thereby quantifying estimation uncertainty [30]. As shown in Figure 4, the GPR fitted means (blue line and scatter points) generally matched the measured values (red squares) across the 75 fitted observations. The blue shaded area represents the 95% observation interval, and 73 of the 75 fitted observations fell within this interval (97.3%). Two observations, Samples 26 and 33, fell above the upper bound of the interval, indicating that the fitted mean underestimated the observed HTS content under these two conditions. In addition, the interval width exhibits variations across the sequence: it is wider at steep peaks and valleys (samples 26–33 and 58–60) and narrower in flat, stable regions (samples 11–20 and 66–70). This indicates that regions with data fluctuations or lower local data density exhibit higher model uncertainty, whereas dense and stable regions show lower uncertainty. Therefore, the wider intervals suggest that targeted data supplementation and further experimental verification are required in these high-uncertainty regions to enhance the model’s reliability.

Other ML analyses (such as XGBoost-GA for exploring model-estimated candidate regions and constraint trade-offs, and RF for feature importance ranking) were performed to illustrate the exploratory capabilities of the two algorithms. Due to their in-sample fitting nature and the lack of experimental validation, these outputs are provided for reference alone. Detailed results are presented in Supplementary Materials (Figures S6–S8 and Tables S14 and S15).

### 2.3. HTS Pretreatment Reduces CAP-Induced Ca^2+^ Influx and MAPK Phosphorylation

**Figure 5:**
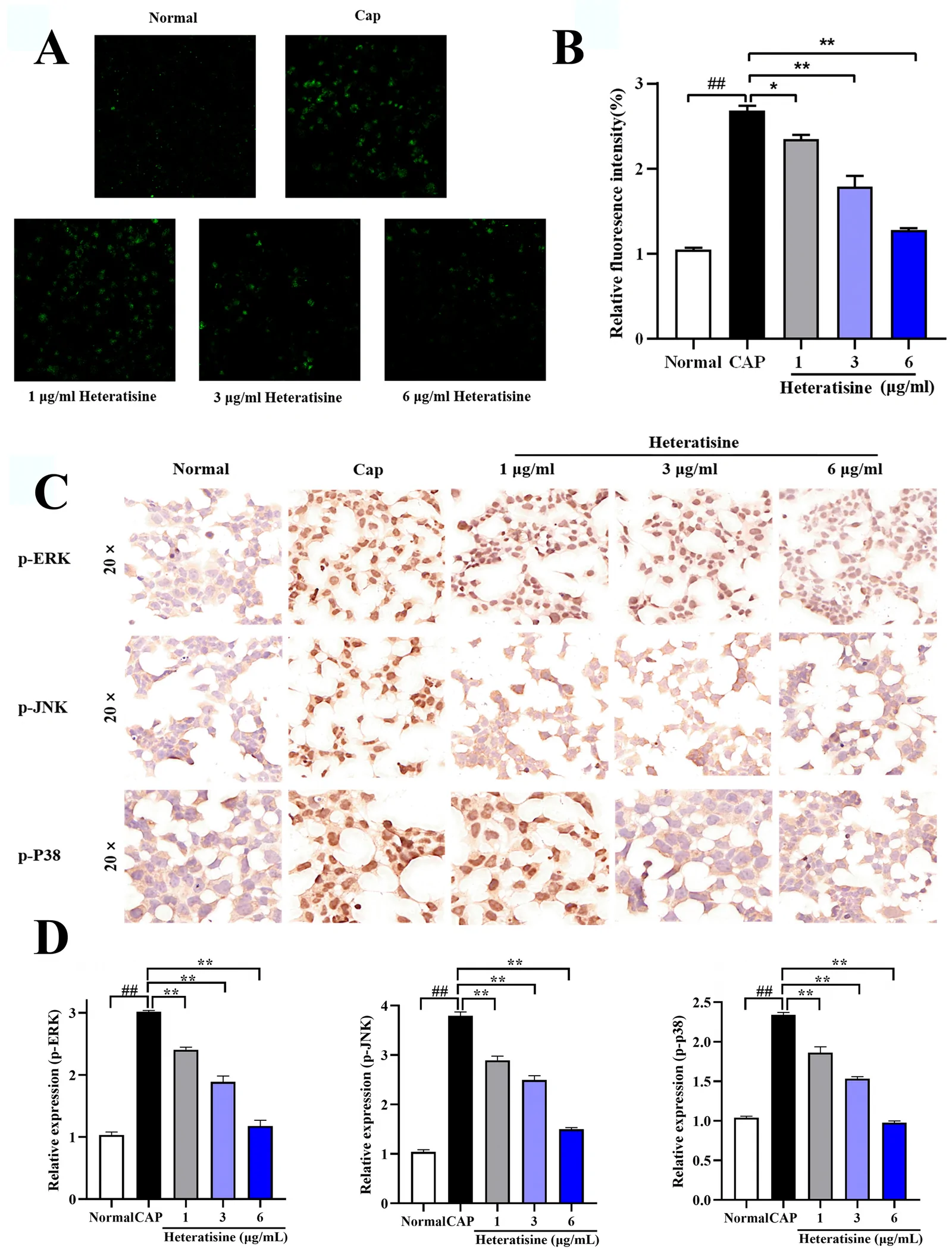
HTS reduces CAP-induced Ca^2+^ influx (**A**,**B**) and phosphorylation of ERK, JNK and p38 (**C**,**D**) in HEK293T-TRPV1 cells. *n* = 3 (biological replicates), ##, *p* < 0.01, with normal control group; *, *p* < 0.05, **, *p* < 0.01, with CAP group.

Within the safe concentration range (≤25 μg/mL in RAW264.7 macrophages and ≤6 μg/mL in HEK293T-TRPV1 cells, Figures S9 and S10), HTS showed weak NO inhibition at 6 μg/mL (15.3 μM), indicating limited anti-inflammatory activity (Figure S11). CAP activates TRPV1 to trigger Ca^2+^ influx and downstream MAPK phosphorylation, a nociceptive signaling cascade in pain transduction [31]. In HEK293T-TRPV1 cells, CAP significantly increased intracellular Ca^2+^ fluorescence, whereas HTS reduced this effect dose-dependently (Figure 5A,B). As MAPK nodes, ERK, JNK, and p38 phosphorylation was also reduced by HTS (Figure 5C,D). Compared with the CAP model group, 1 μg/mL HTS significantly reduced p-ERK, p-JNK, and p-p38 (*p* < 0.05), while 3 and 6 μg/mL induced further reductions (*p* < 0.01). The minimum effective concentration was 1 μg/mL (2.56 μM), lower than that for NO inhibition. These findings suggest that attenuation of TRPV1-mediated signaling may contribute to the potential analgesic activity of HTS and support further investigation of its analgesic mechanisms.

## 3. Discussion

Extraction is a multi-objective task that should balance extraction content, experimental cost, solvent safety, scalability, and environmental impact [32]. In this context, the present study followed a stepwise approach, beginning with solvent screening, followed by condition optimization, and culminating in preparative-scale purification. During the solvent screening stage, the diversity of DES combinations rendered exhaustive experimental screening costly; thus, COSMO-RS was introduced for theoretical pre-screening. The predicted trends across different DES systems and molar ratios were consistent with the experimental contents, indicating that this computational approach effectively narrowed the experimental scope and reduced trial-and-error efforts [33]. It should be noted that COSMO-RS predicted chloroform to have a stronger solvation capacity for HTS than the Men-Oct, corresponding to an approximately 6-fold difference in calculated solubility, whereas the experimental UAE gave only a 1.7-fold difference (Table 1). A similar discrepancy was observed between Thy-Men and EtOH, where the calculated solubility order did not match the experimental contents. This is because COSMO-RS predicts thermodynamic affinity at infinite dilution, whereas actual UAE is a kinetically controlled process influenced by cell-wall penetration, diffusion, solvent viscosity, and mass transfer limitations [34]. COSMO-RS has been reported to overestimate partition ratios and extraction efficiencies, particularly for halogen-containing solvent systems [35], and the quantitative prediction of extraction performance from thermodynamic models alone remains challenging in complex plant-matrix systems [12]. Therefore, while COSMO-RS is a useful tool for qualitative solvent ranking and eliminating candidates, its predictions should be validated experimentally, especially for plant extractions where transport phenomena play a critical role. For hydrophobic DES systems, such as Men-Oct, combine hydrogen-bonding behavior with nonpolar alkyl chains, which favors compatibility with weakly polar HTS. Their immiscibility with water prevents phase separation and maintains a homogeneous extraction phase, and may also facilitate plant-matrix penetration and contact with HTS, although their relatively high viscosity could limit mass transfer, which was mitigated by ultrasound. Solvent recovery was not evaluated because HTS content was determined directly by HPLC in extraction experiments and the DES was separated during chromatography in the preparative procedure, which is a limitation of this study.

In process optimization, modeling requires both estimation accuracy and systematic understanding of the extraction behavior, such as factor contributions and data distribution. Therefore, the choice of modeling should be guided by the actual characteristics of the data, rather than forcing the data to conform to a predetermined model structure [36]. Under standard experimental designs, QPR can establish the functional relationship between factors and response with a limited number of runs and assess factor interactions. Evaluation of the 75-run dataset indicated that QPR revealed the estimation boundary of the conventional approach on unstructured data, while ML extracted additional information from the same dataset. XGBoost, RF, and GPR provide distinct exploratory value by identifying model-estimated candidate regions, ranking joint factor contributions, and locating data-sparse regions for future experiments, respectively. These outputs collectively move beyond a single estimated optimum and provide a more comprehensive basis for data-driven understanding and planning future experimental designs. Due to the limited availability of plant material, the 75 runs were used for model fitting and internal evaluation rather than external validation. Because this dataset consists only of non-structured corner-point combinations with two temperature levels, the identified candidate regions, observation intervals, and factor contributions are merely surrogate model estimates within the observed range. Future experiments with available material could validate these estimates.

HTS exhibited a stronger effect on CAP-induced Ca^2+^ signaling than on NO production, suggesting preferential action on the peripheral nociceptive signaling terminal. TRPV1 integrates nociceptive signals and can be sensitized or activated by inflammatory mediators [37]. Therefore, modulating this channel is considered a potential strategy for pain intervention. The cell results are consistent with previous reports that diterpenoid alkaloids from *Aconitum* plants may modulate the same target [38]. However, these assays do not establish direct TRPV1 antagonism, and the reduced Ca^2+^ signal may also reflect altered TRPV1 expression or localization, calcium homeostasis, or dye loading rather than direct channel inhibition. Future investigations incorporating empty-vector controls, established TRPV1 antagonists, and patch-clamp electrophysiology are necessary to further explore this phenomenon and clarify the direct interaction mechanism.

## 4. Materials and Methods

### 4.1. Materials

All chemical reagents of analytical and HPLC grade were purchased from Sinopharm Co., Ltd. (Shanghai, China). *A. tanguticum* was obtained from Qinghai University and authenticated by Professor Qien Li. A voucher specimen (20230826) was deposited in the Lab for Natural Products Chemistry, School of Pharmacy, South-Central Minzu University. Griess kits (nitric oxide, NO), Lipo8000 transfection reagent and Fluo-4 AM Ca^2+^ fluorescent probe were obtained from Beyotime company (Shanghai, China). Bovine serum albumin (BSA), Triton X-100, paraformaldehyde, 3,3′-diaminobenzidine (DAB), hematoxylin, primary antibodies against phosphorylated extracellular signal-regulated kinase (p-ERK), c-Jun N-terminal kinase (p-JNK), p38 mitogen-activated protein kinase (p-p38), peroxidase (HRP)-conjugated secondary antibodies, thiazolyl blue (MTT), trypsin, penicillin-streptomycin, LPS and phosphate-buffered saline (PBS) were obtained from Servicebio company (Wuhan, China).

RAW264.7 and HEK293T cell lines were purchased from the China Center for Type Culture Collection, Wuhan University (Wuhan, China). Ampicillin and agar were purchased from Gibco (Gibco Co., Ltd., Beijing Company, Beijing, China). Fetal bovine serum (FBS) was purchased from Tianhang Biotechnology Co., Ltd. (Hangzhou, China).

### 4.2. COSMO-RS Modeling

General calculation and simulation steps, including the “Multiple Solutes” and “Activity Coefficient” modules and the corresponding formulas, followed the procedure described in a previous reference [11]. Calculations were performed using COSMOthermX (2019, version 19.0.4) in combination with TmoleX (version 4.5.3N, COSMOlogic GmbH & Co. KG, Leverkusen, Germany). The structures of individual DES molecules were downloaded from PubChem and were first optimized at the BP86/def-TZVP level of theory within the TmoleX computational environment. For HTS, its 2D relative configuration was drawn in the KingDraw structural software (version 5.0, KingDraw Business Corporation, Qingdao, China), and the R/S configuration of all chiral carbons was labeled. The completed structure was imported into TmoleX, where the computational parameters were set to their default values: exchange-correlation functional BP86 with def-TZVP basis set, singlet spin multiplicity, m3 grid size, DFT-D3 (BJ) dispersion correction, 50 SCF convergence cycles, and DIIS damping parameters: start = 4, minimum = 1, and step = 0. The melting point (258 °C) and enthalpy of fusion (44.5 kJ/mol, 113 J/g) of HTS were determined using a NETZSCH DSC 200F3 differential scanning calorimeter (NETZSCH-Gerätebau GmbH, Selb, Germany) and used as input parameters for the COSMO-RS solubility calculations. Water was included in the COSMO-RS calculations for the non-hydrophobic DES systems at the 20% (*w*/*w*) content used experimentally, with the HBA, HBD, and water mole fractions normalized to 1. For the hydrophobic DES systems, water was not included in the calculations.

### 4.3. Preparation of DES Systems

DES systems were prepared at the mature HBA:HBD molar ratios, which have been reported in the literature [39,40]. Water was not added to the hydrophobic DES systems (LA-Men, Thy-Men, and Men-Oct) because they are immiscible with water and prone to phase separation. For other systems, 20% water (*w*/*w*) was added to the mixture to reduce viscosity and enhance mass transfer during extraction. Each mixture was placed in a 150 mL beaker, ultrasonicated at 60 °C for 30 min, and cooled to room temperature. The resulting DES systems were stored for subsequent use.

### 4.4. Ultrasonic DES Extraction of HTS by RSM

Dried and powdered *A. tanguticum* stems were sieved through a 60-mesh screen. A KQ-400KDE ultrasonic machine was used for extraction (KunShan Ultrasonic Instruments Co., Ltd., Kunshan, China). Initial DES screening used a 1:20 g/mL solid–liquid ratio, 50 °C heating temperature, 30 min extraction time, 300 W ultrasonic power and 20% water content. As mentioned in Section 4.3, water was not added for hydrophobic DES systems. After identifying the most suitable DES system, single-factor experiments were conducted using that system to determine the appropriate ranges of extraction parameters. Optimization was carried out using RSM with a BBD. Experimental data were fitted to a QPR model using Design-Expert software (version 13.0, Stat-Ease Inc., Minneapolis, MN, USA). Model adequacy was evaluated using analysis of variance (ANOVA), and the significance of each term was determined. Optimal conditions were estimated by solving the regression equation and experimentally verified.

HTS content served as the response variable for RSM optimization. Ethanol was used as a reference solvent under identical extraction conditions, whereas chloroform served both for standard preparation and as an additional reference solvent. For DES systems containing acids, the pH of the collected extract was adjusted to 9–10 with aqueous ammonia to convert HTS from its salt form to the free molecular form. This step is consistent with the general principle that alkaloids are protonated under acidic conditions and converted to free bases under alkaline conditions [41]. The mixture was extracted with ethyl acetate, and the organic phase was concentrated under reduced pressure. The residue was dissolved in methanol and made up to a final volume of 5 mL to provide an analytical sample.

### 4.5. ML Methods

To evaluate generalization to extraction conditions and to describe the final models, a two-stage procedure was used. First, a condition-grouped nested validation was performed: outer 5-fold × 3 repeats (seeds 42, 43, 44), with 60 runs (20 conditions) used for training and hyperparameter selection and 15 runs (5 conditions) held out for evaluation. All three replicates of each condition were kept in the same fold. The condition intersection between training and test sets was zero. Second, final descriptive models were built on all 75 runs using the same selection rules. Thus, the comparison constitutes an in-sample fitting description and a separate grouped validation, not an external prediction.

#### 4.5.1. XGBoost Modeling and GA Optimization

In XGBoost, the objective function consists of a loss function and a regularization term:

(1) $$\mathrm{Obj}=\sum_{i=1}^{n}l(y_{i},{\hat{y}}_{i})+\sum_{k=1}^{K}\Omega (f_{k})$$

where *l* (*y_i_*, ${\hat{y}}_{i}$) measures the difference between the estimated and observed values, *Ω* (*f_k_*) is the regularization term that controls model complexity, and *K* is the number of trees. During model fitting, the following parameters were optimized: number of estimators (100, 200, 300), maximum tree depth (3, 5, 7), and learning rate (0.03, 0.05, 0.1). Hyperparameters were selected by three-fold grouped cross-validation within the 60 training runs of each outer fold, minimizing the unweighted mean fold MSE. The outer 15 held-out runs were not used for tuning, preprocessing, or fitting. The final XGBoost model used the same search space and a three-fold grouped cross-validation within the 75 runs (seed 20261016) to select its configuration, followed by refitting on all runs. The fitting/validation record counts were 48/27, 51/24 and 51/24, respectively. Ties were resolved by the first configuration in the fixed parameter-grid order.

The fitted XGBoost model served as the fitness function for an exploratory GA search to identify model-estimated candidate regions. The GA parameters were set as follows: 120 generations, population size 50, crossover rate 0.8 and mutation rate 0.2. A BLX-α crossover operator (α = 0.5) was used, which allows sampling outside the parent interval and avoids the boundary limitation of arithmetic crossover. Each generation underwent selection, crossover and mutation to search for the optimal combination of extraction parameters. The model fitting and estimation performance were evaluated using the coefficient of determination (*R*^2^), root mean square error (RMSE), absolute average deviation (AAD) and standard prediction error (SEP). The relevant formulas are as follows:

(2) $$R^{2}=1-\frac{\sum_{i=1}^{n}(Y_{e}-Y_{p}{)}^{2}}{\sum_{i=1}^{n}(Y_{e}-{\bar{Y}}_{e}{)}^{2}}$$

(3) $$\mathrm{RMSE}=\sqrt{\frac{1}{n}\sum_{i=1}^{n}(Y_{p}-Y_{e}{)}^{2}}$$

(4) $$\mathrm{AAD}=\frac{1}{n}\sum_{i=1}^{n}\left|\frac{Y_{p}-Y_{e}}{Y_{e}}\right|\times 100\%$$

(5) $$\mathrm{SEP}=\frac{\mathrm{RMSE}}{{\bar{Y}}_{e}}\times 100\%$$

where n is the number of samples, *Y_e_* is the experimental value, *Y_p_* is the estimated value, and ${\bar{Y}}_{e}$ is the mean experimental value.

#### 4.5.2. RF Modeling with Feature Importance

RF is an ensemble method based on bootstrap aggregation. Hyperparameter selection was performed via three-fold grouped cross-validation within the 60 training runs of each outer fold. For the final model, the same three-fold grouped cross-validation was applied within 75 runs (seed 20261016). The fitting/validation record counts were 48/27, 51/24 and 51/24, and the search space covered the number of trees (50, 150, 300), maximum depth (3, 5, 7, 10), and minimum samples per leaf (1, 2, 4). From the fitting set, B bootstrap samples are drawn with replacement, each used to train a regression tree. At each node split, a random subset of m features is selected as candidates to decorrelate the trees. The final estimate is the average of all tree outputs:

(6) $$\hat{y}(x)=\frac{1}{B}\sum_{b=1}^{B}T_{b}\left(x\right)$$

where *T_b_* is the b-th regression tree. Each tree is grown by recursively minimizing the sum of squared errors at the child nodes:

(7) $$\min_{j,s}\left[\sum_{x_{i}\in R_{1}}{\left(y_{i}-c_{1}\right)}^{2}+\sum_{x_{i}\in R_{2}}{\left(y_{i}-c_{2}\right)}^{2}\right]$$

where *j* is the splitting feature, *s* is the split point, *R*_1_ and *R*_2_ are the resulting left and right regions, and *c*1 and *c*2 are the mean response values within each region.

Feature importance was evaluated using Gini importance (mean decrease in impurity). For each feature, the weighted reduction in squared error across all splits and trees was summed, averaged over trees, and finally normalized so that the importances of the four factors sum to 1. Candidates were scored by negative mean squared error.

#### 4.5.3. GPR Probabilistic Modeling

GPR is a Bayesian nonparametric method that places a Gaussian process prior over the unknown function *f*(*x*) as follows:

(8) $$f(x)\sim \mathrm{GP}\left(m(x),k\left(x,x\prime \right)\right)$$

where *m*(*x*) is the mean function (set to a constant via normalize_y = True), and *k*(*x*, *x*′) is the covariance kernel function. The observation model is *y* = *f*(*x*) + *ε*, where *ε*~*N*(0, $\sigma_{n}^{2}$).

The kernel used in this study was the anisotropic Matérn kernel (*ν* = 5/2) with a constant kernel and a white noise kernel:

(9) $$k\left(x,x\prime \right)=\sigma_{f}^{2}\cdot \left(1+\sqrt{5}r+\frac{5}{3}r^{2}\right)\cdot \exp\left(-\sqrt{5}r\right)+\sigma_{n}^{2}\cdot \delta \left(x,x^{\prime }\right)$$

where *r* =$\sqrt{\sum_{d=1}^{4}{\left(\frac{x_{d}-x_{d}^{\prime }}{l_{d}}\right)}^{2}}$, *l_d_* are the length scales for each input feature, $\sigma_{f}^{2}$ is the signal variance, $\sigma_{n}^{2}$ is the observation noise variance learned from data via the white noise kernel, and *δ* is the Kronecker delta. The *ν* = 5/2 setting ensures twice-differentiable sample paths, which are suitable for modeling smooth extraction response surfaces. Given fitting data (*X*, *y*), the posterior distribution at a new point $x_{*}$ is Gaussian:

(10) $$\mu \left(x_{*}\right)=k_{*}^{T}{\left(K+\sigma_{n}^{2}I\right)}^{-1}y$$

(11) $$\sigma^{2}\left(x_{*}\right)=k\left(x_{*},x_{*}\right)-k_{*}^{T}{\left(K+\sigma_{n}^{2}I\right)}^{-1}k_{*}$$

where K is the covariance matrix of the fitting points, and $k_{*}$ is the covariance vector between the new point and fitting points. The 95% observation interval was taken as $\mu \left(x_{*}\right)\pm 1.96\cdot \sigma (x_{*})$.

Hyperparameters ($\sigma_{f}$, $l_{d}$, and $\sigma_{n}$) were optimized by maximizing the log marginal likelihood:

(12) $$\log p\left(y|X,\theta \right)=-\frac{1}{2}y^{T}{\left(K+\sigma_{n}^{2}I\right)}^{-1}y-\frac{1}{2}\log\left|K+\sigma_{n}^{2}I\right|-\frac{n}{2}\log(2\pi )$$

Hyperparameter optimization was performed using only the 60 training runs of each outer fold. For the final model, the same kernel structure and optimization rules were applied to 75 runs. The four input features were standardized using the training folds only. The length scale search range was 0.1–10, with 20 random restarts of the L-BFGS-B optimizer to avoid local optima. A white noise kernel was included to learn the observation noise level from the data, rather than fixing it to an arbitrarily small value, which would lead to overconfident estimates.

All ML procedures were implemented in Python 3.8 with the XGBoost library (for XGBoost-GA) and scikit-learn (for RF and GPR). The parameter search spaces, fold memberships, selected parameters, preprocessing rules, candidate scores and fixed seeds are summarized in Supplementary Tables S1–S4, S6 and S9. Data processing and visualization were carried out using NumPy (version 1.20), Pandas (version 1.3), and Matplotlib (version 3.3) within the PyCharm environment (version 2023.1.4). The random seed was set to 42 for the outer grouped validation and to 20261016 for the 75-run model selection. The final selection seed was fixed prior to the calculation and was not optimized based on the final in-sample fit scores. A *p*-value < 0.05 was considered statistically significant.

### 4.6. HPLC Analysis of HTS Content

The general procedures were conducted according to a reference [4]. HTS was determined using an Agilent 1200 series HPLC system (Agilent Technologies, Santa Clara, CA, USA), equipped with a quaternary low-pressure pump, column oven, autosampler and diode array detector. Separation was performed on a Cosmosil ODS MS-II C18 column (250 mm × 4.6 mm, 5 μm, Nacalai Tesque Inc., Kyoto, Japan) with isocratic elution of methanol-water (containing 0.1% trifluoroacetic acid, TFA) at a volume ratio of 15:85 at 1.0 mL/min. The column temperature was maintained at 30 °C, and the detection wavelength was 210 nm. The reference standard (5 mg HTS) was dissolved in a small volume of chloroform with ultrasonic assistance, then diluted to 10 mL with methanol in a volumetric flask.

Both standard and sample solutions were filtered through a 0.45 μm membrane before injection. Linearity (injection volumes of 1, 2, 4, 6, 8, 10 and 12 μL), stability, precision, repeatability and spike-and-recovery tests were validated. A calibration curve was constructed by plotting peak area against the corresponding injection volume (or concentration) of the reference standard, and the regression equation was obtained. For ethanol extracts, the HTS standard solution was diluted 10-fold to establish the calibration curve. Each sample powder (2 g) was extracted, partitioned and made up to 5 mL with methanol in a volumetric flask. Low-content samples (EtOH and Thy-Men extracts) were quantified using the diluted calibration curve, and other samples were quantified using the undiluted standard curve. The test samples were injected at a fixed volume of 15 μL, and the on-column content of HTS was determined via the regression equation and confirmed to be within the validated range of the calibration curve. The sample content was expressed as mg/g.

### 4.7. HTS Preparation

Based on the RSM-optimized extraction method, 200 g of *A. tanguticum* powder was extracted under the optimal conditions. After extraction, the crude extract was purified by octadecyl-bonded silica (Nucifera C18, Hanbon Science & Technology Co., Ltd., Huaian, Jiangsu, China) medium-pressure chromatography and eluted with 20% methanol (containing 0.1% TFA, 15 mL/min). HTS was directly isolated by preparative HPLC under the same chromatographic conditions as the analytical method. The preparative system consisted of a Waters 1525 pump (Waters Shanghai Branch Office, Shanghai, China), a 2996 photodiode array detector and a 2487 autosampler. Separation was performed on a Daisogel ODS C18 column (250 mm × 20 mm, 10 μm, Soda Co., Ltd., Osaka, Japan) at a flow rate of 10 mL/min, with detection at 210 nm. The injection volume was 200 μL per run. Prepared HTS was identified by nuclear magnetic resonance spectroscopy (NMR, ^1^H, ^13^C, Figure S1) on a Bruker AVANCE III HD 600 MHz spectrometer (Bruker Corporation, Billerica, MA, USA) and high-resolution mass spectrometry (HR-MS) on a Q-Exactive hybrid quadrupole-Orbitrap mass spectrometer (Thermo Fisher Scientific, Waltham, MA, USA).

### 4.8. Cellular Evaluation of Analgesic Potential

#### 4.8.1. Cell Culture and Cytotoxicity Assay

RAW264.7 and HEK293T cells were maintained in Dulbecco’s Modified Eagle Medium (DMEM, Thermo Fisher Scientific, Waltham, MA, USA) supplemented with 10% FBS and 1% penicillin-streptomycin at 37 °C in a humidified atmosphere of 5% CO_2_. Cells were passaged every 2–3 days and seeded at appropriate densities for experiments.

Both RAW264.7 and HEK293T cells were seeded in 96-well plates at 10^5^ cells/mL. The culture medium was replaced with medium containing HTS at concentrations of 1–200 μg/mL (1, 3, 6, 12, 25, 50, 100 and 200), and incubated for 24 h. The normal control group received an equal volume of complete culture medium. After treatment, MTT solution (0.5 mg/mL) was added and incubated for 4 h. Formazan crystals were dissolved in dimethyl sulfoxide (DMSO), and absorbance was measured at 490 nm. Cell viability was calculated as the percentage of the untreated control. Safe concentrations for cell growth (cell viability > 90%) were selected for subsequent experiments.

#### 4.8.2. LPS-Induced NO Production Assay

RAW264.7 cells were seeded in 6-well plates and treated with HTS (1, 3, 6, 12, 25 μg/mL) for 24 h, followed by stimulation with LPS (1 μg/mL) for 2 h. The culture supernatant was collected, and the level of NO was determined using Griess kits. Absorbance was measured at 540 nm, and concentrations were calculated from standard curves.

#### 4.8.3. CAP-Mediated Ca^2+^ Influx Assay

*Escherichia coli* (*E. coli*) competent cells were thawed on ice, mixed with the TRPV1 plasmid, incubated on ice for 30 min, heat-shocked at 42 °C for 1.5 min, and cooled on ice for 3 min. Lysogeny Broth (LB) medium (Thermo Fisher Scientific, Waltham, MA, USA) was added, and the cells were incubated with shaking for 1 h, then spread onto LB agar plates with ampicillin and cultured overnight at 37 °C. Single colonies were picked, inoculated into LB medium, cultured with shaking for 14 h, and centrifuged at 4000 rpm for 10 min. The pellet was resuspended, and the plasmid was extracted using a plasmid extraction kit according to the manufacturer’s instructions.

For transfection, HEK293T cells were plated overnight. The TRPV1 plasmid, green fluorescent protein (GFP) plasmid, and transfection reagent were mixed in Opti-MEM I reduced-serum (Opti-MEM) medium, incubated for 20 min, and added dropwise to the cells. After 48 h, transfection efficiency was evaluated using confocal microscopy (LSM900, Carl Zeiss AG, Jena, Germany). Cells with 80–85% efficiency were selected for subsequent experiments.

HTS and CAP were dissolved in DMSO (cell culture grade, Servicebio company, Wuhan, China) and diluted in the culture medium. Based on the cytotoxicity results (Section 4.8.1), HTS was used at 1, 3, and 6 μg/mL. CAP was diluted to 8 μM in PBS. HEK293T-TRPV1 cells were pretreated with HTS for 24 h. After the HTS-containing medium was removed, cells were loaded with 2 μM Fluo-4 AM for 30 min at 37 °C in the dark, washed three times with PBS, and stimulated with CAP (except the blank control). Intracellular Ca^2+^ fluorescence was measured within 30 s after CAP stimulation using a confocal microscope and quantified using ImageJ (version 1.54h).

#### 4.8.4. Immunocytochemistry

HEK293T-TRPV1 cells were seeded on glass coverslips in 6-well plates (2 × 10^5^ cells per coverslip). After 4 h, the medium was replaced, and cells were pretreated with HTS (1, 3 and 6 μg/mL) for 24 h, then stimulated with CAP (8 μM) for 30 min. Cells were fixed with 4% paraformaldehyde at room temperature for 20 min, permeabilized with 0.1% Triton X-100 for 10 min, washed with PBS, and blocked with 5% BSA for 30 min. Primary antibodies against p-ERK, p-JNK, and p-p38 (1:500) were applied overnight at 4 °C. After washing, HRP-conjugated secondary antibodies (1:2000) were added for 30 min, followed by DAB staining (5–10 min) and hematoxylin counterstaining. Coverslips were mounted, and images were captured under an optical microscope. Positive staining was quantified with ImageJ based on area and optical density.

### 4.9. Statistical Analyses

All statistical analyses were performed using Origin 2026. Data are presented as mean ± standard deviation (SD). Cellular experiments were performed with three biological replicates. Within each replicate, multiple fields were measured and averaged before analysis. For two-group comparisons, a two-tailed Student’s *t*-test was used. For multi-group comparisons, one-way ANOVA was followed by Dunnett’s multiple comparisons test, using the CAP (model) group as the reference for comparison with other groups, with correction for multiple comparisons. Statistical significance was set at *p* < 0.05. Graphical results were generated using GraphPad Prism (version 10.3).

## 5. Conclusions

In this study, a DES-based UAE process for HTS from *A. tanguticum* was established by integrating COSMO-RS screening, RSM optimization via BBD, and ML evaluation on a 75-run dataset. COSMO-RS proved effective in predicting solute and solvent compatibility and guided the selection of Men-Oct as the optimal DES, which streamlined solvent selection without exhaustive experimentation. RSM optimized the extraction process within the structured BBD design, achieving an HTS content equivalent to 90.7% of that obtained with chloroform. For the non-structured dataset, the ML models demonstrated relatively high in-sample fitting capacity compared to QPR, thereby providing exploratory information and a reference for handling complex data structures. Cell-based assays indicated that HTS attenuated CAP-induced Ca^2+^ signaling and phosphorylation signals. These findings provide a transferable workflow for alkaloid extraction and process development while supporting further investigation of the bioactivity of HTS from *A. tanguticum*.

## Figures and Tables

**Table 1:** Calculated mass fraction solubility and infinite dilution activity coefficient lnγ^∞^ by COSMO-RS and experimental content of HTS in DES systems ^a^.

Extraction System	Mole Ratio	HBD/HBA	Calculated Mass Fraction Solubility ^b^	Calculated ln(γ^∞^)	Experimental Extraction Content mg/g
Bet-PG	1:4	Betaine-Propylene glycol	2.764 × 10^−4^	2.568	− ^c^
	1:5		3.369 × 10^−4^	2.376	−
ChCl-Urea	1:2	Choline chloride-Urea	1.241 × 10^−6^	7.807	−
ChCl-LA	1:1	Choline chloride-Lactic acid	1.96 × 10^−5^	5.084	−
	1:2		3.411 × 10^−5^	4.403	−
	1:3		8.006 × 10^−5^	3.732	−
ChCl-Gly	1:2	Choline chloride-Glycerol	3.044 × 10^−5^	4.512	−
ChCl-PG	1:2	Choline chloride-Propylene glycol	7.065 × 10^−5^	3.711	−
	1:3		1.134 × 10^−4^	3.263	−
	1:4		1.608 × 10^−4^	2.929	−
LA-Gly	1:1	Lactic acid-Glycerol	1.008 × 10^−3^	1.081	−
	1:2		6.276 × 10^−4^	1.554	−
LA-Men	1:2	Lactic acid-Menthol	6.2 × 10^−3^	−1.739	0.106
Thy-Men	1:2	Thymol-Menthol	3.579 × 10^−3^	−1.32	0.062
Men-Oct	1:2	Menthol-Octanoic acid	0.01	−2.342	0.321
EtOH			4.366 × 10^−3^	−0.304	0.035
CHCl_3_			0.06	−4.194	0.55

^a^, Values are means of three technical replicates; ^b^, dimensionless unit; ^c^, −, below the detection limit.

**Table 2:** ANOVA results for ultrasound-assisted HTS extraction.

Source	Sum of Squares	Degree of Freedom	Mean Square	*F*-Value	*p*-Value	
Model	0.1269	14	0.0091	88.64	<0.0001	significant
A-Power	0.0025	1	0.0025	24.59	0.0002	
B-Time	0.0025	1	0.0025	24.06	0.0002	
C-S/L	0.0009	1	0.0009	8.36	0.0118	
D-Temperature	0.0028	1	0.0028	26.99	0.0001	
AB	0.0028	1	0.0028	27.75	0.0001	
AC	0.0012	1	0.0012	11.85	0.0040	
AD	0.0017	1	0.0017	16.57	0.0011	
BC	0.0000	1	0.0000	0.1588	0.6962	
BD	0.0003	1	0.0003	2.89	0.1112	
CD	0.0010	1	0.0010	9.51	0.0081	
A^2^	0.0249	1	0.0249	243.30	<0.0001	
B^2^	0.0585	1	0.0585	571.89	<0.0001	
C^2^	0.0317	1	0.0317	309.82	<0.0001	
D^2^	0.0546	1	0.0546	534.06	<0.0001	
Residual	0.0014	14	0.0001			
Lack of Fit	0.0012	10	0.0001	2.51	0.1944	not significant
Pure Error	0.0002	4	0.0000			
Cor Total	0.1283	28				

## Data Availability

Data is contained within the article or Supplementary Materials.

## Supplementary Materials

The following supporting information can be downloaded at https://www.mdpi.com/article/10.3390/plants15213362/s1, Figure S1: HR-ESI-MS, ^1^H and ^13^C NMR data for HTS; Figures S2 and S3: σ-profile and σ-potential of HTS; Figure S4: σ-potential of HTS, ChCl, and Urea; Figure S5: HPLC chromatograms of HTS from DES extractions; Figure S6: Genetic algorithm convergence and repeated runs on the fitted XGBoost surrogate; Figure S7: RF feature importance on the 75-run dataset; Figure S8: Fixed-condition slices comparing QPR and XGBoost at a ≥0.42 mg/g threshold; Figures S9 and S10: RAW264.7 cell viability and HEK293T-TRPV1 cell viability with HTS; Figure S11: NO production in LPS-stimulated RAW264.7 cells treated with HTS; Table S1: In-sample diagnostics of XGBoost configurations; Tables S2–S4: Hyperparameter configurations of XGBoost-GA optimization, RF modeling and GPR modeling; Table S5: RSM experiment design for HTS extraction; Table S6: Hyperparameters and configurations for the 29-run dataset under the unified data-adaptive selection rules; Table S7: Fitting performance of four modeling approaches on the 29-run dataset; Table S8: The 75 dataset in QPR and ML comparison; Table S9: Hyperparameters and configurations for the 75-run dataset under the unified data-adaptive selection rules; Table S10: Evaluation metrics of the four models on the 75 dataset; Table S11: Fixed-parameter sensitivity check: applying 75-run configurations to the 29-run dataset; Table S12: Outer fold split audit for the condition-grouped nested validation; Table S13: Repeated condition-grouped nested validation of the modeling procedures; Table S14: Comparison of feasible region coverage between XGBoost and QPR; Table S15: Model estimates under different engineering constraints.
