# Supplementary material for: Deep Eutectic Solvent Extraction of Heteratisine from Aconitum tanguticum: COSMO-RS Screening, RSM Optimization and Machine Learning Comparison
Source: Plants (Basel). 2026 Oct 29;15(21):3362. doi: 10.3390/plants15213362 (PMC13765846; doi:10.3390/plants15213362)
Supplement: Supplementary file 1 [file plants-15-03362-s001.zip › plants-4606345-supplementary.pdf]

Deep Eutectic Solvent Extraction of Heteratisine from *Aconitum tanguticum*: COSMO-RS Screening, RSM Optimization and Machine Learning Comparison

Ruixi Gao [1], Haishuo Huang [1], Xinyang Li [1], Junjie Ma [1], Jiawei Dai [1], Yuchen Lei [1], Liqun Ma [2], Awais Ihsan [3], Qien Li [4], Jun Li [1], Qupei Danzeng [5,*], Xianju Huang [1,6,*]

[1] *School of Pharmaceutical Sciences, South-Central Minzu University, Wuhan, China*

[2] *College of Life Sciences, South-Central Minzu University, Wuhan, China*

[3] *Department of Biosciences, COMSATS University Islamabad Sahiwal Campus, Sahiwal, Pakistan*

[4] *Tibetan Medical College, Qinghai University, Xining, China*

[5] *Department of Tibetan Pharmacy, Xizang University of Tibetan Medicine, Lhasa, China*

[6] *Hubei International Science and Technology Cooperation Base (SH2311), South-Central Minzu University, Wuhan, China*

Qupei Danzeng

hellodz@hotmail.com

Xianju Huang

xianju@mail.scuec.edu.cn

Contents

Supplementary figures	Name	Page
Figure S1.	HR-ESI-MS, ^{1}H and ^{13}C NMR data for HTS	3
Figure S2.	σ-profile of HTS	4
Figure S3.	σ-potential of HTS	4
Figure S4.	σ-potential of HTS, ChCl and Urea	5
Figure S5.	HPLC chromatograms of HTS from DES extractions	6
Figure S6.	Genetic algorithm convergence and repeated runs on the fitted XGBoost surrogate	6
Figure S7.	RF feature importance on the 75-run dataset	7
Figure S8.	Fixed-condition slices comparing QPR and XGBoost at a ≥ 0.42 mg/g threshold	7
Figure S9.	RAW264.7 cell viability with HTS	8
Figure S10.	HEK293T-TRPV1 cell viability with HTS	8
Figure S11.	NO production in LPS-stimulated RAW264.7 cells treated with HTS	8
**Supplementary tables**		
Table S1.	In-sample diagnostics of XGBoost configurations	9
Table S2.	Hyperparameter configuration of XGBoost-GA optimization	9
Table S3.	Hyperparameter configuration of RF modeling	10
Table S4.	Hyperparameter configuration of GPR modeling	10
Table S5.	RSM experiment design for HTS extraction	11
Table S6.	Hyperparameters and configurations for the 29-run dataset under the unified data-adaptive selection rules	12
Table S7.	Fitting performance of four modeling approaches on the 29-run dataset	12
Table S8.	The 75 dataset for QPR and ML comparison	13
Table S9.	Hyperparameters and configurations for the 75-run dataset under the unified data-adaptive selection rules	15
Table S10.	Evaluation metrics of the four models on the 75 dataset	15
Table S11.	Fixed-parameter sensitivity check: applying 75-run configurations to the 29-run dataset	16
Table S12.	Outer fold split audit for the condition-grouped nested validation	16
Table S13.	Repeated condition-grouped nested validation of the modeling procedures	17
Table S14.	Comparison of feasible region coverage between XGBoost and QPR	17
Table S15.	Model estimates under different engineering constraints	17

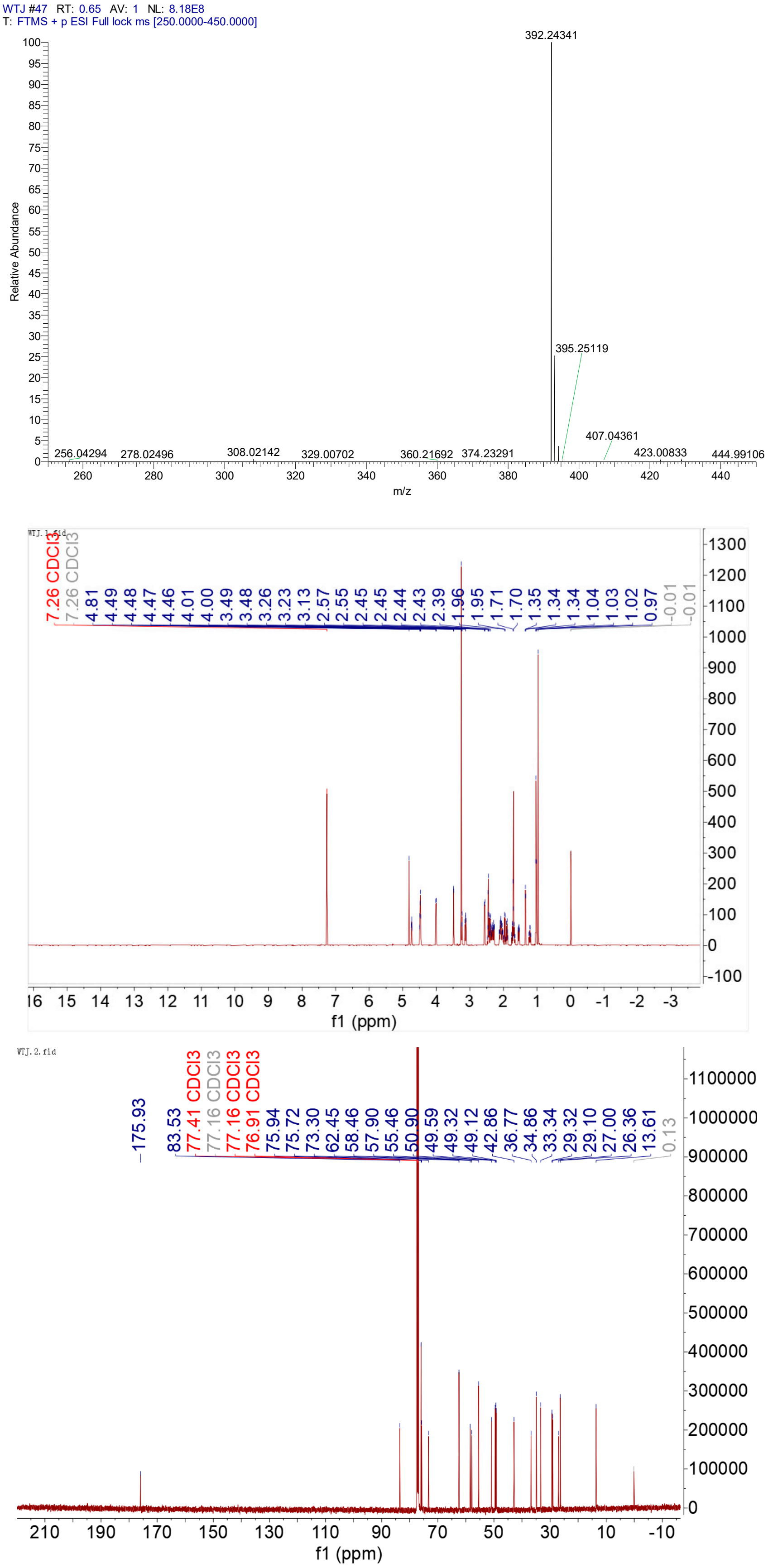

Figure S1. HR-ESI-MS (positive mode), ^{1}H and ^{13}C NMR of HTS (in $CDCl_3$).

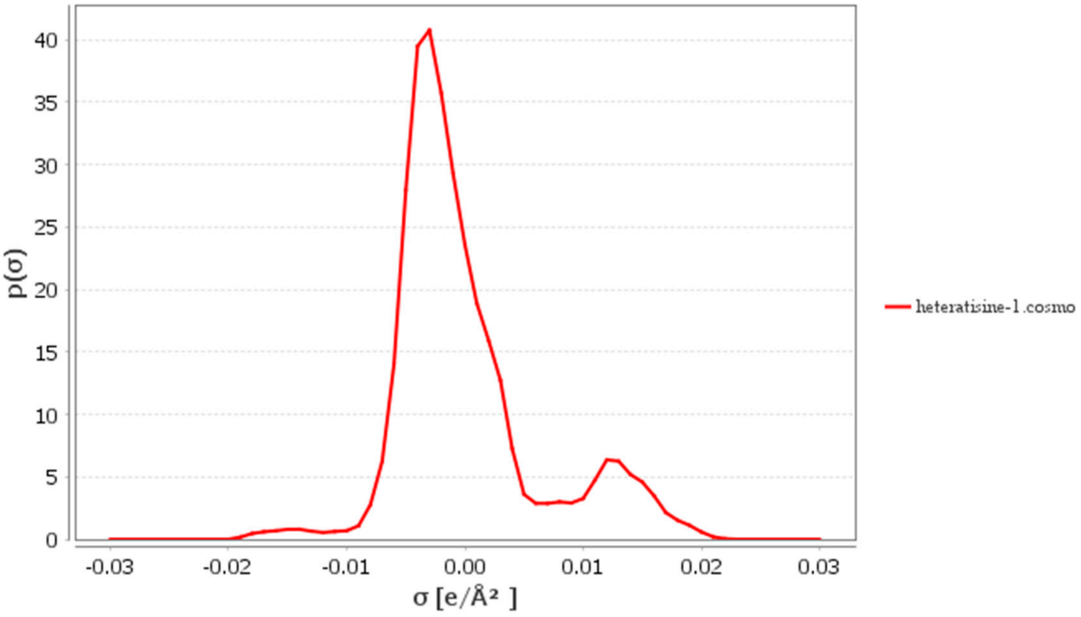

Figure S2. σ-profile of HTS.

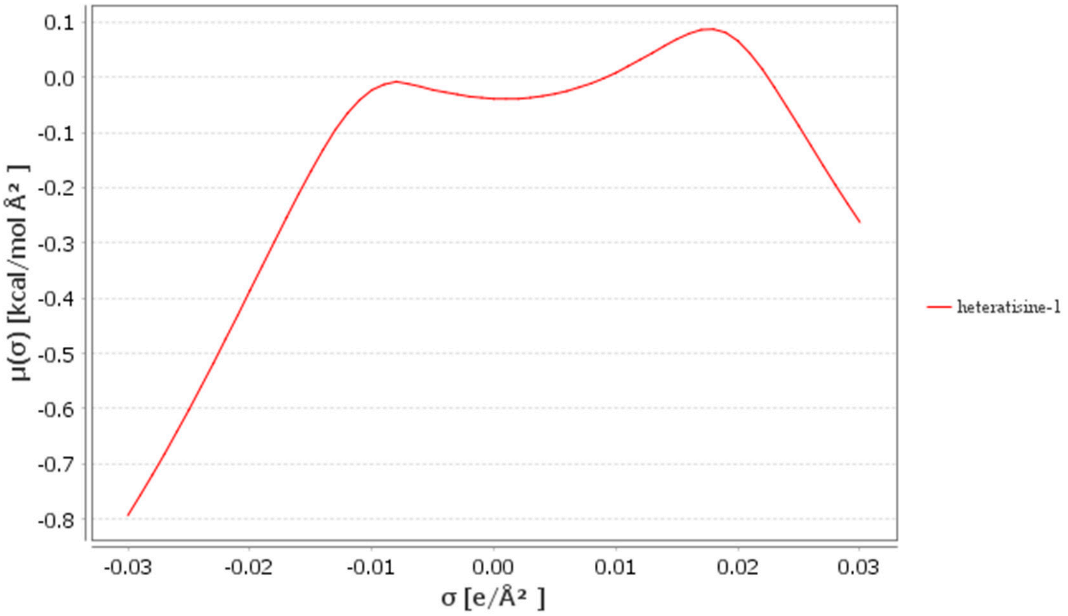

Figure S3. σ-potential of HTS.

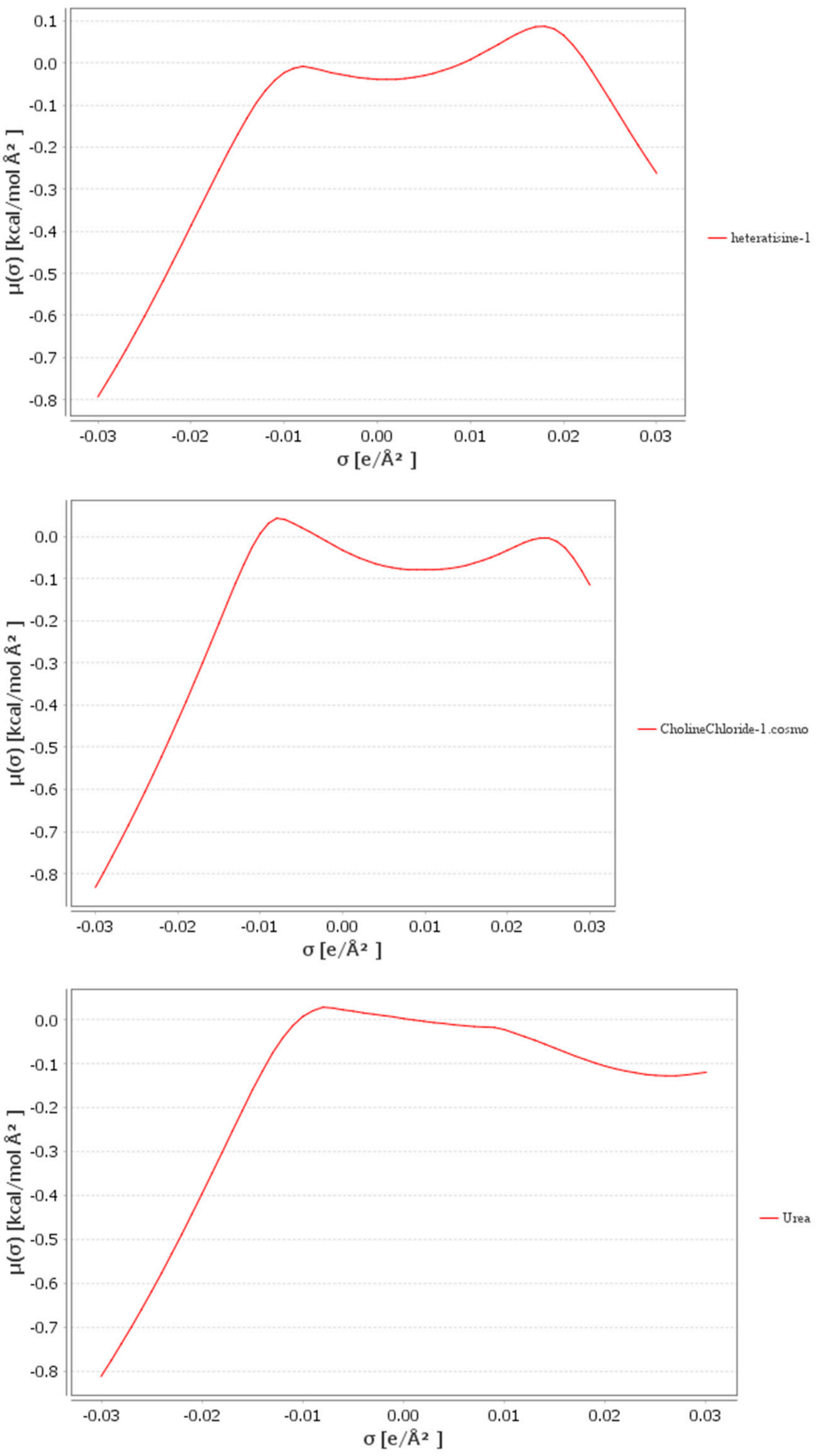

Figure S4. σ-potential of HTS, ChCl and Urea.

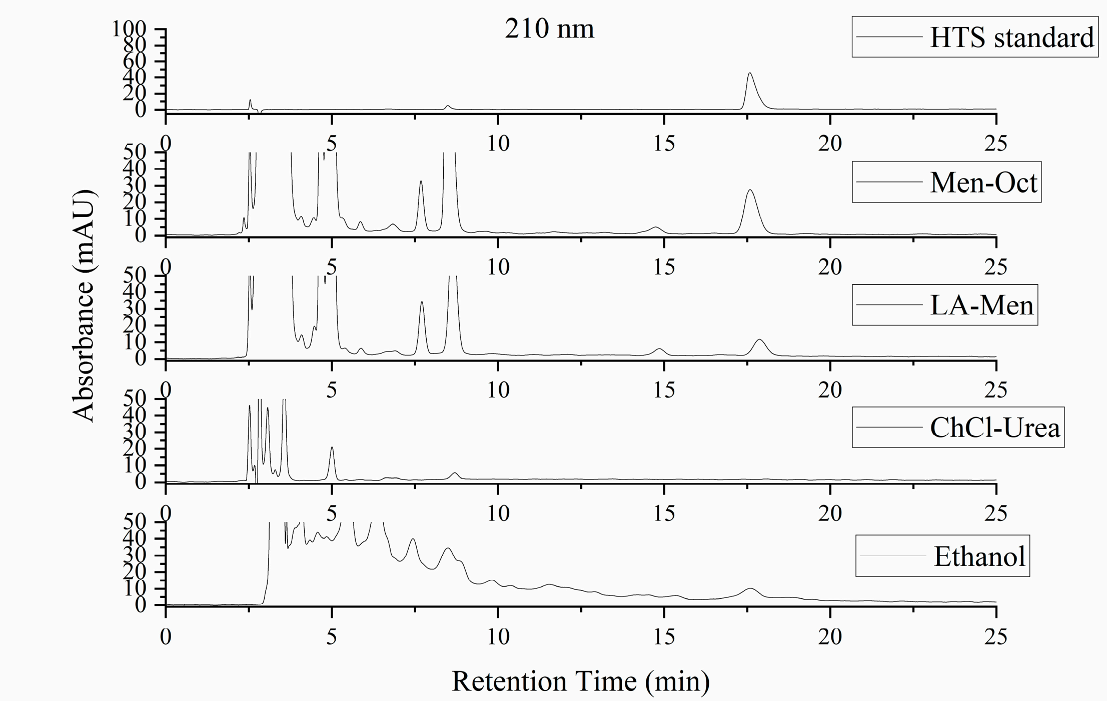

Figure S5. HPLC chromatograms of HTS from DES extractions. Men-Oct, menthol-octanoic acid, LA-Men, lactic acid-menthol, ChCl-Urea, choline chloride-urea.

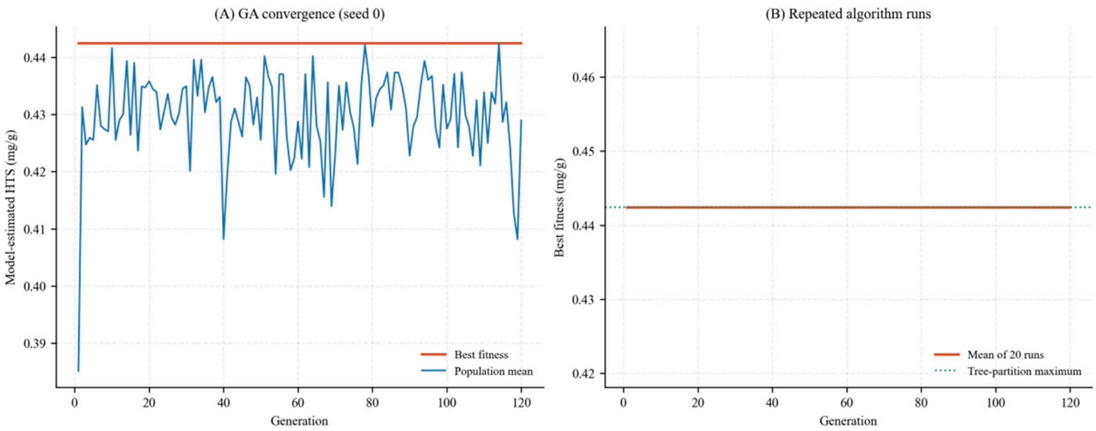

Figure S6. Genetic algorithm (GA) convergence and repeated runs on the fitted XGBoost surrogate. A, single GA run (seed 0); B, mean of 20 runs vs. exhaustive tree-partition maximum.

The piecewise constant surface of XGBoost causes the best fitness to plateau immediately at 0.442 mg/g. The 20-run mean matches the exhaustive maximum, confirming GA reproducibility. This plateau is a mathematical boundary of the model, not a validated physical optimum.

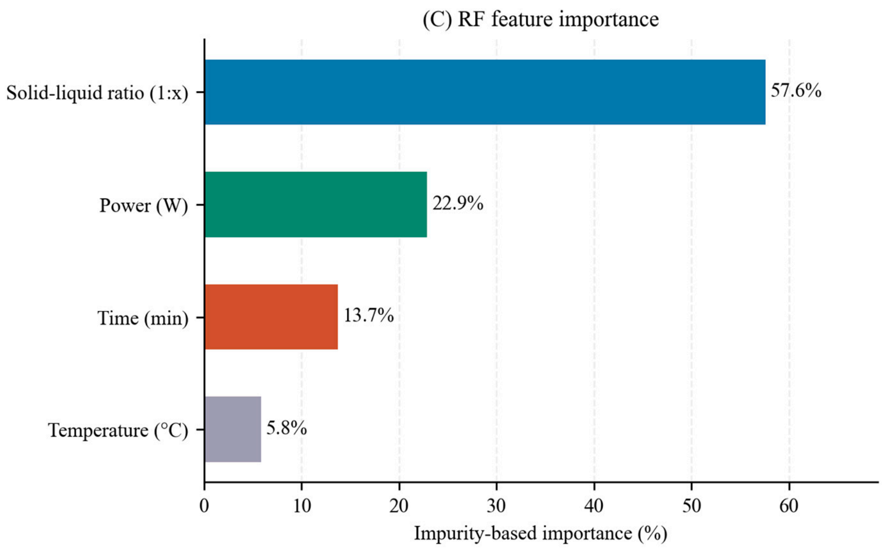

Figure S7. RF feature importance on the 75-run dataset.

Solid-liquid ratio shows the highest impurity-based importance (57.6%), followed by power (22.9%), time (13.7%), and temperature (5.8%). Given the restricted tree depth used to prevent overfitting in this non-structured data (R^2 = 0.546), this ranking serves only as an exploratory description of the model's internal splitting behavior and requires independent experimental validation.

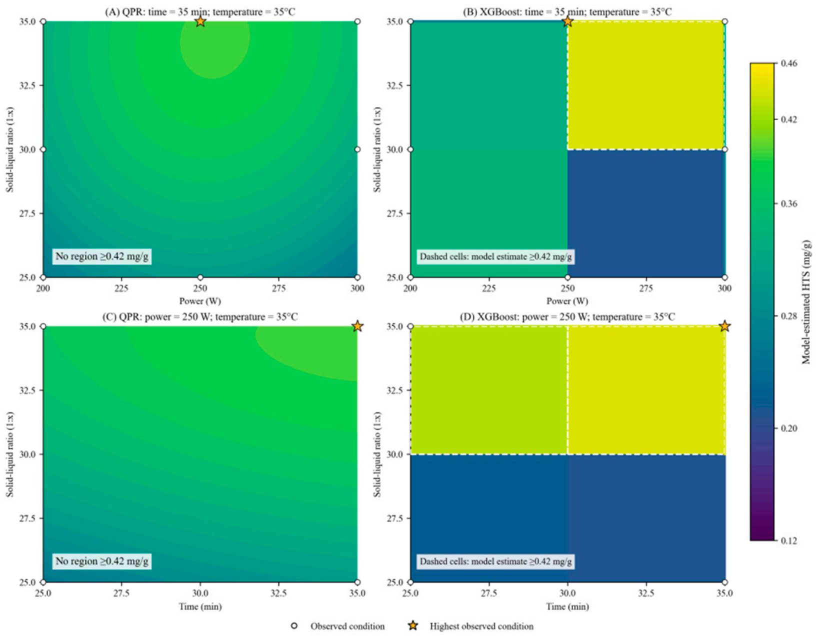

Figure S8. Fixed-condition slices comparing QPR and XGBoost at a ≥ 0.42 mg/g threshold.

QPR shows smooth gradients with no qualifying regions, whereas XGBoost identifies distinct model-estimated regions (yellow dashed cells), reflecting its piecewise constant nature. These regions are exploratory model estimates and have not been experimentally validated as stable operating windows.

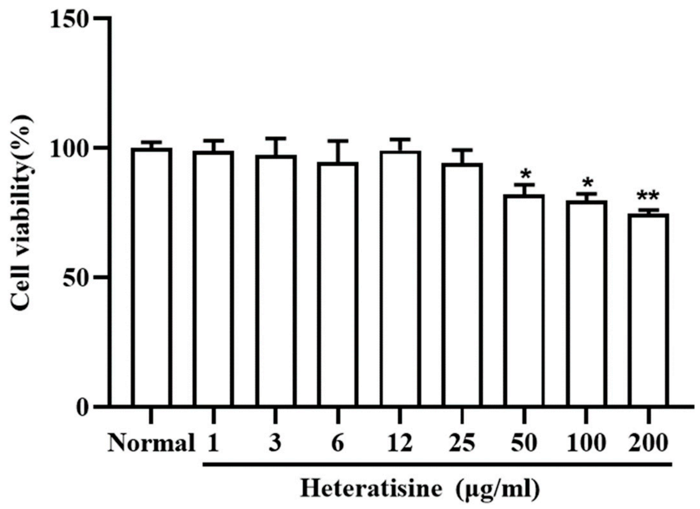

Figure S9. RAW264.7 cell viability after treatment with HTS. n=5 (biological replicates); *, $p \leqq 0.05$, **, $p \leqq 0.01$.

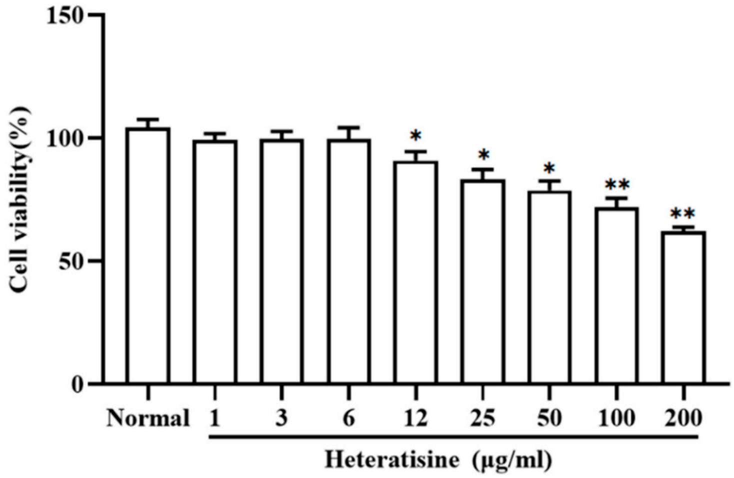

Figure S10. HEK293T-TRPV1 cell viability after treatment with HTS. n = 5 (biological replicates); *, $p \leqq 0.05$, **, $p \leqq 0.01$.

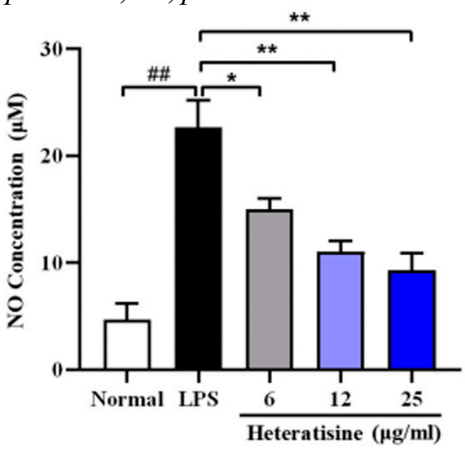

Figure S11. NO production in LPS-stimulated RAW264.7 cells treated with HTS. n = 5 (biological replicates); ##, $p \leqq 0.01$; *, $p \leqq 0.05$, **, $p \leqq 0.01$.

Table S1. In-sample diagnostics of XGBoost configurations.

**Configuration**	**Fitting MSE (mg/g)²**	**Fitting R^2**	**Selected**
n=50, d=3, lr=0.1	0.000897683	0.820088	No
n=100, d=3, lr=0.1	0.000392735	0.921289	No
n=100, d=5, lr=0.1	2.98924e-05	0.994009	No
n=150, d=5, lr=0.05	2.99825e-05	0.993991	No
n=200, d=5, lr=0.05	2.99823e-05	0.993991	No
n=200, d=7, lr=0.05	2.95448e-05	0.994079	No
n=300, d=5, lr=0.05	2.99823e-05	0.993991	No
n=300, d=7, lr=0.03	2.95519e-05	0.994077	No
n=100, d=5, lr=0.03	8.31831e-05	0.983329	Yes

Table S2. Hyperparameter configuration of XGBoost-GA optimization.

Parameter	Value	Description
**XGBoost model parameters**		
n_estimators	100	Number of boosting rounds
max_depth	5	Maximum tree depth
learning_rate	0.03	Step size shrinkage
random_state	42	Random seed for reproducibility
**GA parameters**		
Population size	50	Number of individuals per generation
Generations	120	Maximum number of generations
Crossover rate	0.8	Probability of crossover
Mutation rate	0.2	Probability of mutation
Crossover operator	BLX-α (α = 0.5)	Blend crossover allowing sampling outside parent interval
Selection method	Tournament (size 3)	Selection pressure for reproduction
Elitism	2	Number of best individuals preserved per generation
random_state	0–19	Random seed for reproducibility

Table S3. Hyperparameter configuration of RF modeling.

Parameter	Value	Description
n_estimators	150	Number of trees
max_depth	3	Maximum tree depth
min_samples_leaf	4	Minimum samples per leaf node
min_samples_split	2	Minimum samples to split an internal node
max_features	'sqrt'	Number of features considered at each split ($\sqrt{4} = 2$)
bootstrap	True	Sampling with replacement
criterion	squared_error	Splitting criterion
random_state	42	Random seed for reproducibility

Table S4. Hyperparameter configuration of GPR modeling.

Parameter	Value	Description
Kernel function	ConstantKernel × Matérn + WhiteKernel	$\nu = 2.5$; four standardized inputs
Signal variance	1.024319	Standardized target scale
Length scales	0.588954, 1.208263, 0.100000, 0.109943	Power, time, ratio, temperature; standardized inputs
White noise variance	0.008760419	Standardized target scale
White noise SD	0.006611 mg/g	Restored original response units
Log marginal likelihood	−0.214945	Optimized objective function value
Length-scale bounds	0.1–10	Sensitivity results in Table S14
Signal/noise variance bounds	10^{-3}–10^{3} / 10^{-8}–1	ConstantKernel / WhiteKernel
alpha	10^{-6}	Additional training diagonal variance on normalized scale
normalize_y/StandardScaler	True/used	Fitted using the 75 primary records
Optimizer restarts/random state	20/42	L-BFGS-B marginal likelihood optimization

Table S5. RSM experiment design for HTS extraction.

Run	A/Power (W)	B/Time (min)	C/Solid/Liquid ratio	D/Temperature (°C)	HTS Content (mg/g)
1	200	25	30	40	0.324
2	300	25	30	40	0.301
3	200	35	30	40	0.302
4	300	35	30	40	0.386
5	250	30	25	35	0.346
6	250	30	35	35	0.343
7	250	30	25	45	0.273
8	250	30	35	45	0.332
9	200	30	30	35	0.362
10	300	30	30	35	0.334
11	200	30	30	45	0.301
12	300	30	30	45	0.356
13	250	25	25	40	0.312
14	250	35	25	40	0.336
15	250	25	35	40	0.321
16	250	35	35	40	0.338
17	200	30	25	40	0.344
18	300	30	25	40	0.353
19	200	30	35	40	0.327
20	300	30	35	40	0.405
21	250	25	30	35	0.309
22	250	35	30	35	0.325
23	250	25	30	45	0.262
24	250	35	30	45	0.313
25	250	30	30	40	0.477
26	250	30	30	40	0.492
27	250	30	30	40	0.489
28	250	30	30	40	0.494
29	250	30	30	40	0.491

Table S6. Hyperparameters and configurations for the 29-run dataset under the unified data-adaptive selection rules.

Model	Hyperparameters/Configuration
QPR	Complete quadratic basis: intercept + 4 linear terms + 4 squared terms + 6 interaction terms; 15 columns, rank 15; ordinary least squares fitting.
XGBoost	n_estimators = 300; max_depth = 3; learning_rate = 0.05. (Fixed: random_state = 42, tree_method = hist, objective = reg:squarederror).
RF	n_estimators = 150; max_depth = 7; min_samples_leaf = 1. (Fixed: max_features = sqrt, min_samples_split = 2, bootstrap = True, random_state = 42).
GPR	Input: StandardScaler, normalize_y = True. Kernel: ConstantKernel × Matérn(ν = 2.5) + WhiteKernel. Optimization: alpha = 1e-6, L-BFGS-B, 20 restarts, random_state = 42.
GPR fitted kernel values	Signal variance = 0.579524116; Length scales = [1.036146502, 0.714878732, 5.405242054, 0.654647547]; White noise variance = 0.009889418 (normalized response scale); Log marginal likelihood = −22.877606860.

Table S7. Fitting performance of four modeling approaches on the 29-run dataset.

Model	R^2	RMSE (mg/g)	MAE (mg/g)	AAD (%)	SEP (%)
QPR (RSM)	0.989	0.007	0.006	1.672	1.959
XGBoost	0.998	0.003	0.002	0.492	0.900
RF	0.969	0.012	0.010	3.022	3.308
GPR	0.998	0.003	0.001	0.372	0.749

Table S8. The 75-run dataset for QPR and ML comparison.

Run	A/Power (W)	B/Time (min)	C/Solid/Liquid ratio (g/mL)	D/Temperature (°C)	HTS Content (mg/g)
1	250	35	25	35	0.198
2	250	35	35	35	0.46
3	200	30	35	35	0.354
4	200	25	35	40	0.262
5	200	30	35	35	0.352
6	200	35	25	35	0.348
7	300	30	35	35	0.304
8	300	25	30	35	0.298
9	200	35	25	40	0.244
10	300	35	25	35	0.259
11	250	35	25	35	0.211
12	200	25	35	35	0.385
13	200	30	25	35	0.211
14	200	30	35	35	0.351
15	300	25	25	35	0.171
16	300	25	25	35	0.17
17	200	35	30	35	0.338
18	300	30	25	35	0.236
19	200	35	30	35	0.331
20	200	35	25	40	0.246
21	200	35	35	40	0.248
22	200	25	35	35	0.389
23	300	25	35	35	0.232
24	300	30	35	35	0.302
25	300	35	35	35	0.35
26	300	25	30	35	0.315
27	300	25	30	35	0.285
28	250	25	25	35	0.213
29	250	35	35	35	0.46
30	250	25	25	35	0.215
31	300	25	35	35	0.23
32	250	25	25	35	0.216
33	300	35	25	35	0.296
34	200	25	25	40	0.264
35	250	25	35	35	0.439
36	200	25	35	40	0.263
37	300	35	35	35	0.352
38	200	35	35	35	0.254

Continued

39	300	25	35	35	0.229
40	200	25	25	35	0.294
41	300	30	35	35	0.299
42	250	25	35	35	0.434
43	200	30	25	35	0.222
44	200	25	25	35	0.296
45	200	25	35	40	0.26
46	300	35	30	35	0.329
47	200	35	25	35	0.345
48	200	35	25	40	0.248
49	200	30	30	40	0.329
50	200	35	35	40	0.26
51	300	25	25	35	0.172
52	200	25	30	35	0.226
53	300	30	25	35	0.233
54	200	25	30	35	0.235
55	200	25	30	35	0.223
56	300	35	25	35	0.26
57	200	30	25	35	0.226
58	200	35	25	35	0.341
59	250	35	35	35	0.462
60	300	30	25	35	0.235
61	200	25	35	35	0.383
62	300	35	30	35	0.331
63	200	35	35	40	0.271
64	250	35	25	35	0.205
65	200	25	25	40	0.262
66	250	25	35	35	0.437
67	200	25	25	35	0.297
68	200	35	35	35	0.257
69	300	35	35	35	0.349
70	200	25	25	40	0.266
71	200	30	30	40	0.335
72	200	30	30	40	0.34
73	300	35	30	35	0.321
74	200	35	35	35	0.259
75	200	35	30	35	0.329

Table S9. Hyperparameters and configurations for the 75-run dataset under the unified data-adaptive selection rules.

Model	Predefined Rules/Search Space	Selected Configuration
QPR	Fixed quadratic basis; ordinary least squares	14 columns, rank 13; no parameter search (D^2 omitted due to collinearity)
XGBoost	Trees: 100/200/300; Depth: 3/5/7; LR: 0.03/0.05/0.1	100 trees; Depth 5; LR 0.03
RF	Trees: 50/150/300; Depth: 3/5/7/10; Min leaf: 1/2/4	150 trees; Depth 3; Min leaf 4
GPR	Constant × Matérn(ν=2.5) + WhiteKernel; 20 restarts	Length bounds: 0.1–10; main rules unchanged
GPR fitted kernel values	(Standardized inputs, normalize_y = True; alpha = 1e-6)	Signal variance = 1.024319141; Length scales = [0.588954500, 1.208263206, 0.100000000, 0.109943356]; White noise variance = 0.008760419 (normalized); White noise SD = 0.006611404 mg/g

Notes:

1. Selection Criterion: RF and XGBoost minimized the unweighted mean MSE across 3-fold grouped validation. Ties were resolved by the first configuration in the fixed parameter grid.

2. Fixed Settings: RF used max_features=sqrt, min_samples_split=2, bootstrap=True, criterion=squared_error; XGBoost used objective=reg:squarederror, tree_method=hist. Both used random_state=42.

3. Data Splits: Final 75-run internal 3-fold splits were 48/27, 51/24, and 51/24. All splits kept complete condition groups together (condition intersection = 0).

4. Seed: Final model selection used a fixed seed of 20261016, determined prior to calculation and not optimized based on final fit scores.

Table S10. Evaluation metrics of the four models on the 75-run dataset.

Model	R^2	RMSE (mg/g)	MAE (mg/g)	AAD (%)	SEP (%)
QPR	0.529	0.048	13.773	0.038	16.642
XGBoost	0.983	0.009	2.622	0.007	3.130
RF	0.546	0.047	14.103	0.039	16.336
GPR	0.994	0.005	1.207	0.003	1.855

Table S11. Fixed-parameter sensitivity check: applying 75-run configurations to the 29-run dataset.

Model	Applied 75-run Configuration	R^2	RMSE (mg/g)
XGBoost	100 trees; Depth 5; LR 0.03	0.968	0.012
RF	150 trees; Depth 3; Min leaf 4	0.331	0.054

Table S12. Outer fold split audit for the condition-grouped nested validation.

Repeat	Seed	Fold	Train	Testing	Training Cond.	Testing Cond.	Overlap	Training Record IDs	Testing Record IDs
1	42	1	60	15	20	5	0	3,4,5,6,7,8,10,12,13,14,15,16,17,19,21,22,24,25,26,27,28,30,32,33,34,35,36,37,38,40,41,42,43,44,45,46,47,49,50,51,52,54,55,56,57,58,61,62,63,65,66,67,68,69,70,71,72,73,74,75	1,2,9,11,18,20,23,29,31,39,48,53,59,60,64
1	42	2	60	15	20	5	0	1,2,4,6,7,8,9,10,11,12,13,18,20,21,22,23,24,26,27,28,29,30,31,32,33,34,35,36,38,39,40,41,42,43,44,45,46,47,48,50,52,53,54,55,56,57,58,59,60,61,62,63,64,65,66,67,68,70,73,74	3,5,14,15,16,17,19,25,37,49,51,69,71,72,75
1	42	3	60	15	20	5	0	1,2,3,4,5,6,9,10,11,13,14,15,16,17,18,19,20,23,25,28,29,30,31,32,33,34,35,36,37,38,39,42,43,45,46,47,48,49,51,52,53,54,55,56,57,58,59,60,62,64,65,66,68,69,70,71,72,73,74,75	7,8,12,21,22,24,26,27,40,41,44,50,61,63,67
1	42	4	60	15	20	5	0	1,2,3,4,5,6,7,8,9,10,11,12,14,15,16,17,18,19,20,21,22,23,24,25,26,27,28,29,30,31,32,33,34,36,37,39,40,41,44,45,47,48,49,50,51,53,56,58,59,60,61,63,64,65,67,69,70,71,72,75	13,35,38,42,43,46,52,54,55,57,62,66,68,73,74
1	42	5	60	15	20	5	0	1,2,3,5,7,8,9,11,12,13,14,15,16,17,18,19,20,21,22,23,24,25,26,27,29,31,35,37,38,39,40,41,42,43,44,46,48,49,50,51,52,53,54,55,57,59,60,61,62,63,64,66,67,68,69,71,72,73,74,75	4,6,10,28,30,32,33,34,36,45,47,56,58,65,70
2	43	1	60	15	20	5	0	1,2,3,4,5,6,7,8,9,10,11,12,14,15,16,18,20,22,23,24,25,26,27,29,31,33,35,36,37,38,39,40,41,42,44,45,46,47,48,49,51,52,53,54,55,56,58,59,60,61,62,64,66,67,68,69,71,72,73,74	13,17,19,21,28,30,32,34,43,50,57,63,65,70,75
2	43	2	60	15	20	5	0	1,4,6,7,9,10,11,12,13,15,16,17,18,19,20,21,22,23,24,28,30,31,32,33,34,35,36,38,39,40,41,42,43,44,45,46,47,48,49,50,51,53,56,57,58,60,61,62,63,64,65,66,67,68,70,71,72,73,74,75	2,3,5,8,14,25,26,27,29,37,52,54,55,59,69
2	43	3	60	15	20	5	0	1,2,3,4,5,8,9,10,11,12,13,14,15,16,17,18,19,20,21,22,23,25,26,27,28,29,30,31,32,33,34,35,36,37,39,40,42,43,44,45,48,50,51,52,53,54,55,56,57,59,60,61,63,64,65,66,67,69,70,75	6,7,24,38,41,46,47,49,58,62,68,71,72,73,74
2	43	4	60	15	20	5	0	1,2,3,5,6,7,8,10,11,12,13,14,17,18,19,21,22,24,25,26,27,28,29,30,32,33,34,37,38,40,41,43,44,46,47,49,50,52,53,54,55,56,57,58,59,60,61,62,63,64,65,67,68,69,70,71,72,73,74,75	4,9,15,16,20,23,31,35,36,39,42,45,48,51,66
2	43	5	60	15	20	5	0	2,3,4,5,6,7,8,9,13,14,15,16,17,19,20,21,23,24,25,26,27,28,29,30,31,32,34,35,36,37,38,39,41,42,43,45,46,47,48,49,50,51,52,54,55,57,58,59,62,63,65,66,68,69,70,71,72,73,74,75	1,10,11,12,18,22,33,40,44,53,56,60,61,64,67
3	44	1	60	15	20	5	0	2,3,4,5,6,8,10,12,13,14,15,16,17,18,19,21,22,25,26,27,28,29,30,32,33,34,35,36,37,38,42,43,45,46,47,49,50,51,52,53,54,55,56,57,58,59,60,61,62,63,65,66,68,69,70,71,72,73,74,75	1,7,9,11,20,23,24,31,39,40,41,44,48,64,67
3	44	2	60	15	20	5	0	1,2,3,5,6,7,9,11,13,14,15,16,17,19,20,21,23,24,25,28,29,30,31,32,34,35,37,38,39,40,41,42,43,44,46,47,48,49,50,51,52,54,55,57,58,59,62,63,64,65,66,67,68,69,70,71,72,73,74,75	4,8,10,12,18,22,26,27,33,36,45,53,56,60,61
3	44	3	60	15	20	5	0	1,3,4,5,6,7,8,9,10,11,12,13,14,15,16,18,20,22,23,24,25,26,27,31,33,34,35,36,37,39,40,41,42,43,44,45,46,47,48,49,51,52,53,54,55,56,57,58,60,61,62,64,65,66,67,69,70,71,72,73	2,17,19,21,28,29,30,32,38,50,59,63,68,74,75
3	44	4	60	15	20	5	0	1,2,3,4,5,7,8,9,10,11,12,14,17,18,19,20,21,22,23,24,26,27,28,29,30,31,32,33,34,35,36,38,39,40,41,42,44,45,46,48,49,50,53,56,59,60,61,62,63,64,65,66,67,68,70,71,72,73,74,75	6,13,15,16,25,37,43,47,51,52,54,55,57,58,69

Table S13. Repeated condition-grouped nested validation of the modeling procedures.

Model	$R^2 \pm SD$	RMSE mg/g	MAE mg/g
QPR	-1.432 ± 0.407	0.11	0.088
XGBoost	-0.280 ± 0.253	0.08	0.069
RF	-0.068 ± 0.028	0.073	0.063
GPR	-0.233 ± 0.203	0.078	0.066

Table S14. Comparison of feasible region coverage between XGBoost and QPR.

Content threshold (mg/g)	XGBoost feasible region (% of search space)	QPR feasible region
≥ 0.44	13.066	none
≥ 0.42	24.943	none
≥ 0.40	24.943	none

Table S15. Model estimates under different engineering constraints.

Constraint(s)	Power (W)	Time (min)	Solid/liquid ratio (g/mL)	Tempera ture (°C)	Estimated content (mg/g)	Loss relative to unconstrained
None	275.0	32.5	1:32.5	37.5	0.442	0
Time ≤ 30 min	275.0	30.0	1:32.5	37.5	0.442	0
Power ≤ 250 W	250.0	32.5	1:32.5	37.5	0.442	0
Power ≤ 250 W and time ≤ 30 min	250.0	30.0	1:32.5	37.5	0.442	0
Solid/liquid ratio ≤ 1:30	275.0	32.5	1:30.0	37.5	0.442	0
